# Supplementary material for: Early auto‐immune targeting of photoreceptor ribbon synapses in mouse models of multiple sclerosis
Source: EMBO Mol Med. 2018 Sep 28;10(11):e8926. doi: 10.15252/emmm.201808926 (PMC6220320; doi:10.15252/emmm.201808926)
Supplement: Supplementary file 1 — Appendix [file EMMM-10-e8926-s001.pdf]

Appendix File

Early auto-immune targeting of photoreceptor ribbon synapses in mouse models of multiple sclerosis

Mayur Dembla[1*], Ajay Kesharwani[1], Sivaraman Natarajan[1#], Claudia Fecher-Trost[2], Richard Fairless[3], Sarah K. Williams[3], Veit Flockerzi[2], Ricarda Diem[3], Karin Schwarz[1*], and Frank Schmitz[1*]

[1]Institute of Anatomy and Cell Biology, Department of Neuroanatomy, Saarland University, Medical School, 66421 Homburg, Germany; [2]Institute of Experimental and Clinical Pharmacology and Toxicology, Saarland University, Medical School, 66421 Homburg, Germany; [3]Department of Neurology, University Clinic Heidelberg, Im Neuenheimer Feld 400, 69120 Heidelberg, Germany

* correspondence

Table of content

1.) Appendix Figure S1, including figure legend (page 2)
2.) Appendix Figure S2, including figure legend (page 3-4)
3.) Appendix Figure S3, including figure legend (page 5-6)
4.) Appendix Figure S4, including figure legend (page 7)
5.) Appendix Figure S5, including figure legend (page 8)
6.) Appendix Figure S6, including figure legend (page 9-10)
7.) Appendix Figure S7, including figure legend (page 11-12)
8.) Appendix Figure S8, including figure legend (page 13-14)
9.) Appendix Figure S9, including figure legend (page 15)
10.) Appendix Figure S10, including figure legend (page 16)
11.) Appendix Figure S11, including figure legend (page 17-18)
12.) Appendix Figure S12, including figure legend (page 19-20)
13.) Appendix Figure S13, including figure legend (page 21)
13.) Appendix Figure S14, including figure legend (page 22)
13.) Appendix Figure S15, including figure legend (page 23)

Appendix_Figure_S1

CASPR1(NP_001193824)

```
MMRLRLFCIL LAAVSGARGW GYYGCDEELV GPLYARSLGA SSYYGLFTAP RFARLHGISG    60
WSPRIGDPNP WLQIDLMKKH RIRAVATQGS FNSWDWVTRY MLLYGDRVDS WTPFYQRGHN   120
ATFFGNVNES AVVRHDLHYH FTARYIRIVP LAWNPRGKIG LRLGLYGCPY KSDVLYFDGD   180
DAISYRFPRG VSRSLWDVFA FSFKTEEKDG LLLHAEGAQG DYVTLELQGA HLLLHMSLGS   240
SPIQPRPGHT TVSAGGVLND QHWHYVRVDR FGREANLTLD GYVQRFVLNG DFERLNLDNE   300
MFIGGLVGAA QKNLAYRHNF RGCIENVIFN RVNIADLAVR RHSRITFEGK VAFRCLDPVP   360
HPINFGGPHN FVQVPGFPRR GRLAVSFRFR TWDLTGLLLF SSLGDGLGHV ELMLSEGQVN   420
VSVVQTGRKK LQFAAGFRLN DGFWHEVNFV AQENHAVISI DDVEGAEVRV SYPLLIRTGT   480
SYFFGGCPKP ASRSGCHSNQ TAFHGCMELL KVDGQLVNLT LVEGRRLGYY AEVLFDTCGI   540
TDRCSPNMCE HDGRCYQSWD DFICYCELTG YKGETCHQPL YKESCEAYRL SGKTSGNFTI   600
DPDGSGPLKP FVVYCDIREN RAWTVVRHDR LWTTRVTGSS MERPFLGAVQ YWNASWEEVS   660
ALANASQHCE QWIEFSCYNS RLLNTAGGYP YSFWIGRNEE QHFYWGGSQP GIQRCACGLD   720
RSCVDPALHC NCDADQPQWR TDKGLLTFVD HLPVTQVVVG DTNRSSSEAQ FFLRPLRCYG   780
DRNSWNTISF HTGAALRFPP IRANHSLDVS FYFRTSAPSG VFLENMGGPY CQWRRPYVRV   840
ELNTSRDVVF AFDVGNGDEN LTVHSDDFEF NDDEWHLVRA EINVKQARLR VDHRPWVLRP   900
MPLQTYIWLE YDRPLYVGSA ELKRRPFVGC LRAMRLNGVT LNLEGRANAS EGTSPNCTGH   960
CAHPRFPCFH GGRCVERYSY YTCDCDLTAF DGPYCNHDIG GFFEPGTWMR YNLQSALRSA  1020
AREFSHMLSR PVPGYEPGYI PGYDTPGYVP GYHGPGYRLP DYPRPGRPVP GYRGPVYNVT  1080
GEEVSFSFST QSAPAVLLYV SSFVRDYMAV LIKEDGTLQL RYQLGTSPYV YQLTTRPVTD  1140
GQPHSVNITR VYRNLFIQVD YFSLTEQKFS LLVDSQLDSP KALYLGRVME TGVIDPEIQR  1200
YNTPGFSGCL SGVRFNNVAP LKTHFRTPRP MTEELAEALR VQGELSESNC GAMPRLVSEV  1260
PPELDPWYLP PDFPYYHDDG WVAILLGFLV AFLLLGLVGM LVLFYLQNHR YKGSYHTNEP  1320
KATHDYHAGS KPPLPTSGPV PAPAPASAPT PTPASTQVPA PAPAPAPAPA PGPRDQNLPQ  1380
ILEESRSE                                                          1388
```

CNTN1(NP_776705.1)

```
MKMWLLFSLL VIISFKTCLS EFTWHRRYGH GVSEEDKGFG PIFEEQPINT IYPEESPEGK    60
VSLNCRARAS PFPVYKWRMN NGDIDLTSDR YSMVGGNLVI NNPDKQKDAG IYYCLASNNY   120
GMVRSTEATL SFGYLDPFPP EERPEVRVKE GKGMVLLCDP PYHFPDDLSY RWLLNEFPVF   180
ITMDKRRFVS QTNGNLYIAN VEASDKGNYS CFVSSPSITK SVFSKFIPLI PLPERTTKPY   240
PADIVVQFKD VYALMGQNVT LECFALGNPV PDIRWRKVLE PMPSTAEIST SGAVLKIFNI   300
QLEDEGIYEC EAENNRGKDK HQARIYVQAF PEWVEHINDT EVDIGSDLYW PCVATGKPIP   360
TIRWLKNGYS YHRGELRLYD VTFENAGMYQ CIAENTHGAI YANAELKILA LAPTFEMNPM   420
KKKILAAKGG RVIIECKPKA APKPTFLWSK GTERLVNSSR ILIWEDGSLE INNITRSDGG   480
VYTCFVENNK GKANSTGTLV ITDPTRIILA PINADITVGE NATMQCAASF DPALDLTFVW   540
SFNGYVIDFN KENIHYQRNF MLDSNGELLI RNAQLKHAGR YTCTAQTIVD NSSASADLVV   600
RGPPGPPGGL RIEDIRATSV ALTWSRGSDN HSPISKYTIQ TKTILSDDWK DAKTDPPIIE   660
GNMEAARAVD LIPWMEYEFR VVATNTLGIG EPSIPSNKIK TDGAAPNVAP SDVGGGGGSN   720
RELTITWAPL SREYHYFNNF GYIVAFKPFD GEEWKKVTVT NPDTGRYVHK DETMRPSTAF   780
QVKVKAFNNK GDGPYSLTAV IHSAQDAPSE APTAVGVKVL SSSEISVHWE HVVEKIVESY   840
QIRYWASHDK EAAAHRVQVA SQEYSARLEN LLPDTQYFVE VRACNSAGCG PPSDMTETFT   900
KKAPPSQPPR IISSVRSGSR YIITWDHVVA LSNESTVTGY KVLYRPDGQH DGKLYSTHKH   960
SIEVPIPRDG EYVVEVRAHS DGGDGVVSQV KISGASILSP CLLGFLLPAL GILVYLEF   1118
```

Figure legend: Appendix Figure S1

CASPR1 and CNTN1 peptides identified by mass spectrometry are highlighted in red (24% coverage for CASPR1; 30% coverage for CNTN1). Depicted are the bovine CASPR1 (Contactin associated protein-1, accession numbers NP_001193824 (NCBI) and F1MJQ2 (UniProt); also dubbed CNTNAP1 or Paranodin) and CNTN1 (Contactin1, accession numbers NP_776705.1 (NCBI) or Q28106 (UniProt); also dubbed Neural cell surface protein F3) protein sequences.

Appendix Figure S2

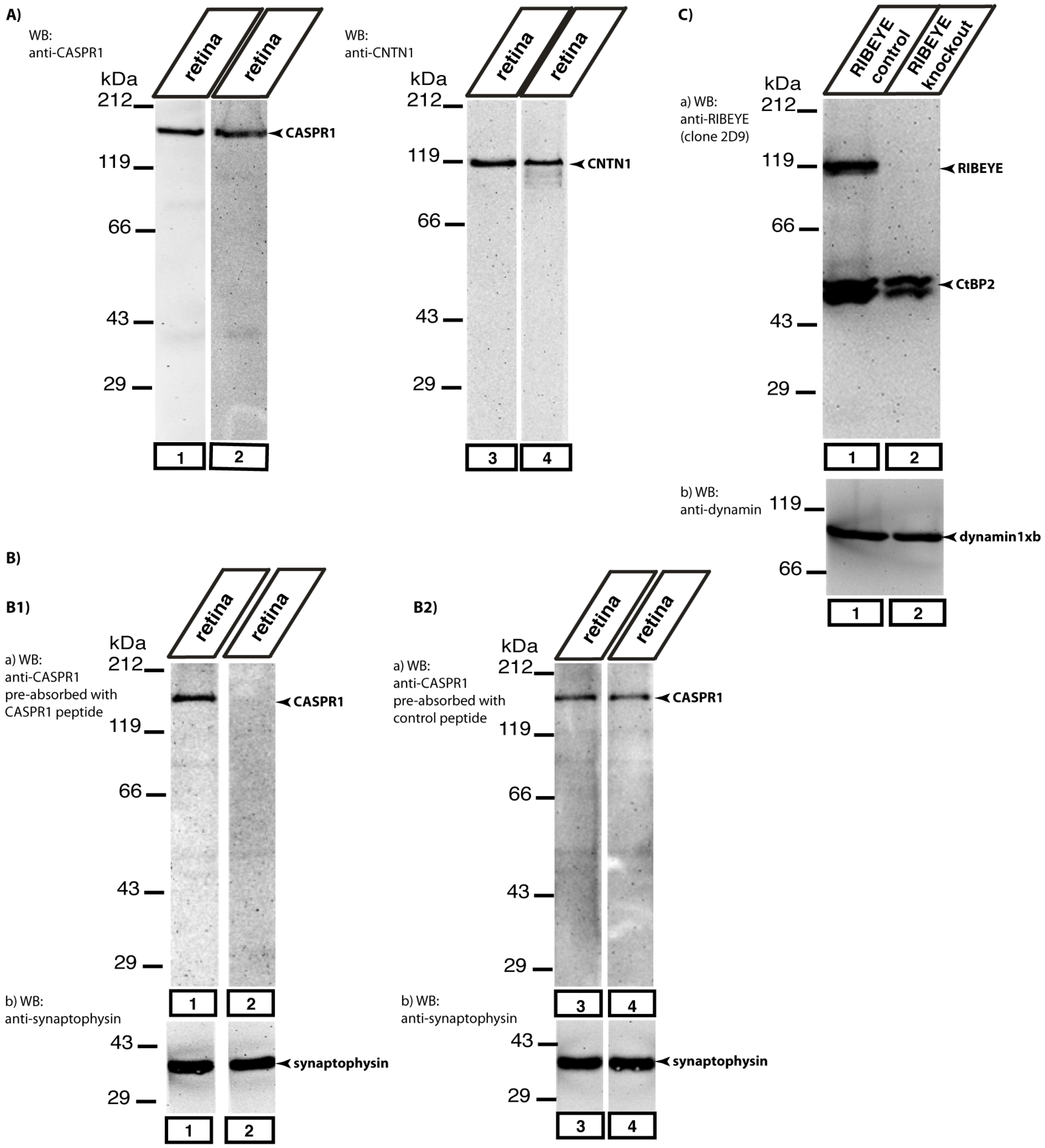

Figure legend: Appendix Figure S2

Mouse retinal lysates were probed with the indicated antibodies for Western blot. (A) Two different independent antibodies against CASPR1 detect a single protein band at the expected running position of CASPR1 at 180kDa (lanes 1,2). Similarly, two independent antibodies against contactin-1 (CNTN1) detect a single protein band at the expected running position of CNTN1 at 120kDa (lanes 3,4). (B) shows a control incubation in which the polyclonal CASPR1 antibody was pre-absorbed either with the CASPR1 peptide against which it was generated (B1, lane 2) or pre-absorbed with a control peptide (B2, lane 4). If the polyclonal CASPR1 antibody was pre-absorbed with the CASPR1 peptide, the 180kDa band was completely abolished (Appendix Fig. S2, B1, lane 2), while the 180kDa band was completely unaffected if the antibody was pre-absorbed with a control peptide (Appendix Fig. 2, B2, lane 4).

In lane 1 (in B1) and lane 3 (in B2), the unblocked CASPR1 antibody was applied for comparison. Immunodetection of synaptophysin served as loading control. (C) Retina tissue from control mice (lane 1) and RIBEYE knockout mice (lane 2) were probed by the monoclonal antibody 2D9 against RIBEYE in Western blot. The ≈120kDa RIBEYE band was completely abolished in the RIBEYE knockout. As expected, CtBP2 is still present in the RIBEYE knockout tissue (Maxeiner et al., 2016). Probing the same nitrocellulose membrane with antibodies against dynamin1xb (Appendix Fig. 2Cb) served as loading control. WB, Western blot.

Appendix Figure S3

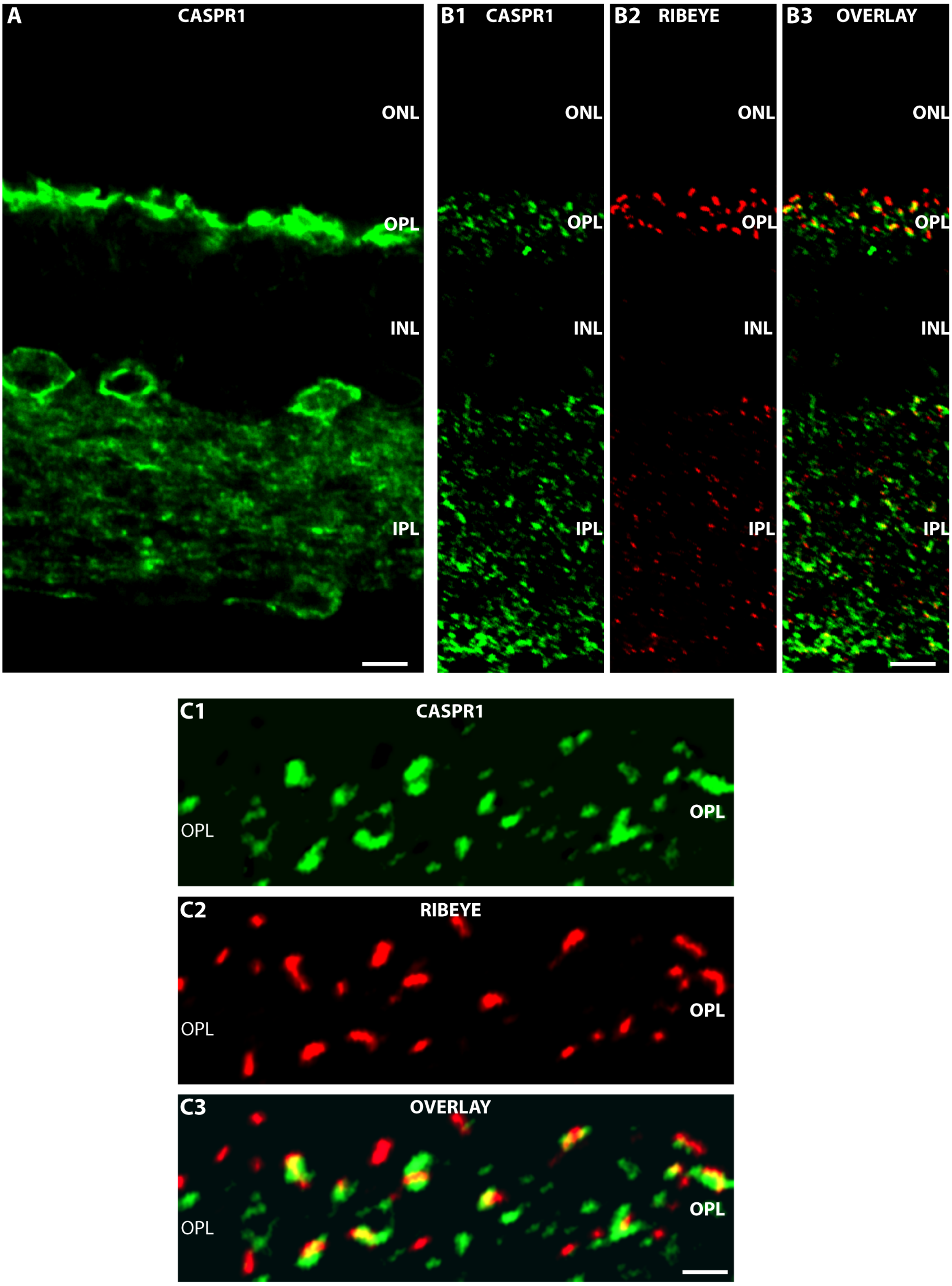

Figure legend: Appendix Figure S3

Cryostat section (A; 10μm in thickness) and semi-thin section (B,C; 0.5μm in thickness) immunolabelled with the indicated antibodies. The cryostat section in A) was immunolabelled with the rabbit polyclonal antibody against CASPR1. In (B,C). the retina was double-immunolabelled with rabbit polyclonal CASPR1 antibody and mouse monoclonal antibody 2D9 against RIBEYE. CASPR1 is strongly expressed in

the synaptic layers of the retina, the OPL and IPL, respectively. High magnification analyses revealed that the CASPR1 immunosignal was particularly enriched in close vicinity to the synaptic ribbon (C). Appendix Fig. 3 was obtained by confocal microscopy. Abbreviations: ONL, outer nuclear layer; OPL, outer plexiform layer; INL, nuclear layer; IPL, inner plexiform layer. Scale bars: 20μm (A,B); 1μm (C).

Appendix Figure S4

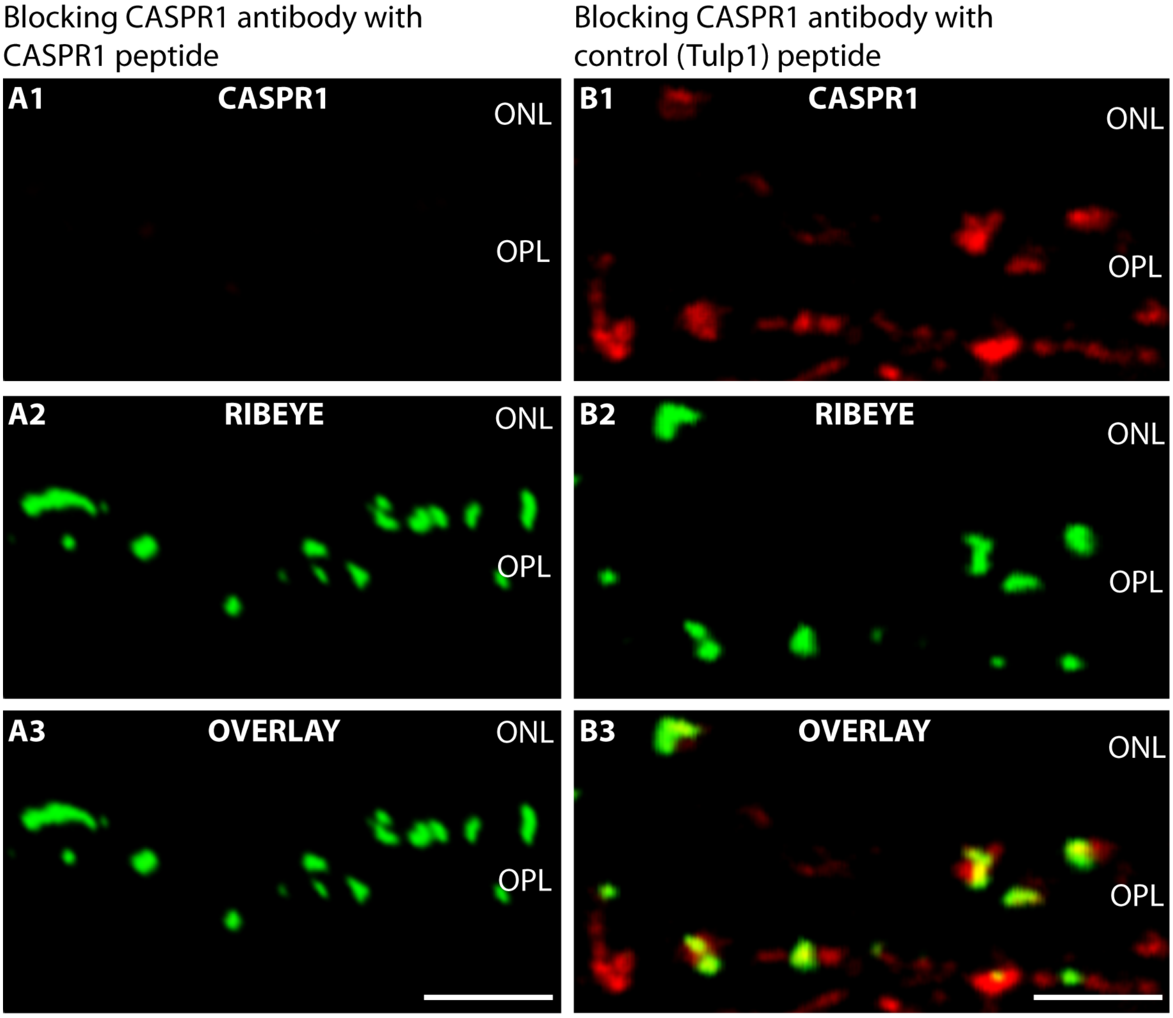

Figure legend: Appendix Figure S4

Semi-thin (0.5μm-thin) sections of the mouse retina immunolabelled with the rabbit polyclonal CASPR1 antibody that was pre-absorbed either with a control peptide (peptide from the Tulp1 protein; Wahl et al., 2016) (B) or with the CASPR1 peptide against which the polyclonal CASPR1 antibody was raised (A). Sections were double-immunolabelled with anti-RIBEYE antibodies to visualize synaptic ribbons. The strong CASPR1 immunosignal in the OPL is completely absent if the rabbit polyclonal CASPR1 antibody was pre-absorbed by the CASPR1 peptide (A) whereas the synaptic CASPR1 immunolabel is completely unaffected if a control peptide was used for pre-absorption (B). The RIBEYE immunolabelling was unaffected by both of these treatments. Appendix Fig. S4 was obtained by confocal microscopy. ONL, outer nuclear layer; OPL, outer plexiform layer. Scale bars: 5μm.

Appendix Figure S5

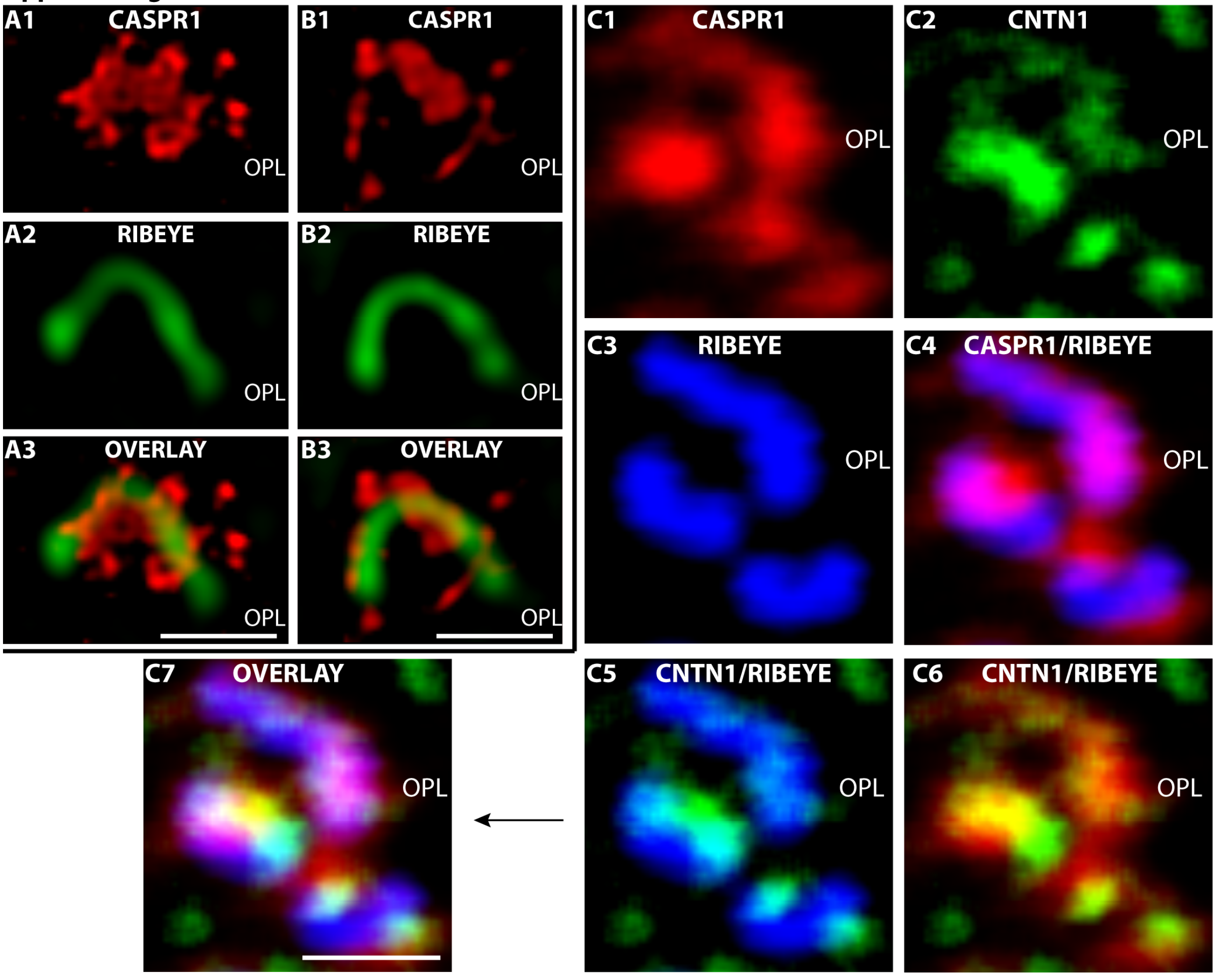

Figure legend: Appendix Figure S5

(A,B) High-resolution analysis of semi-thin sections of the mouse retina double-immunolabelled with the rabbit polyclonal antibody against CASPR1 and the mouse monoclonal antibody against RIBEYE (2D9) obtained by super-resolution structured-illumination-microscopy (SR-SIM). The CASPR1 immunosignal was particularly enriched around the synaptic ribbon. OPL, outer plexiform layer. (C) Contactin-1 (CNTN1) co-localizes with CASPR1 at photoreceptor synapses in the OPL in close vicinity to the synaptic ribbon. High-resolution confocal analyses of rod photoreceptor synapses of the mouse retina (0.5μm sections) triple-immunolabelled with rabbit polyclonal antibody against CASPR1, mouse monoclonal antibody against CNTN1 and rabbit polyclonal antibodies against RIBEYE (U2656). CNTN1 largely has a similar distribution as CASPR1 in photoreceptor synapses of the OPL and is highly enriched at the synaptic ribbon (see also Appendix Fig. S9). In (C4-C6), two immunosignals indicated in the respective figures were overlayered; In C7, all three immunosignals were overlayered to each other. Scale bars: 1μm (A-C).

Appendix Figure S6

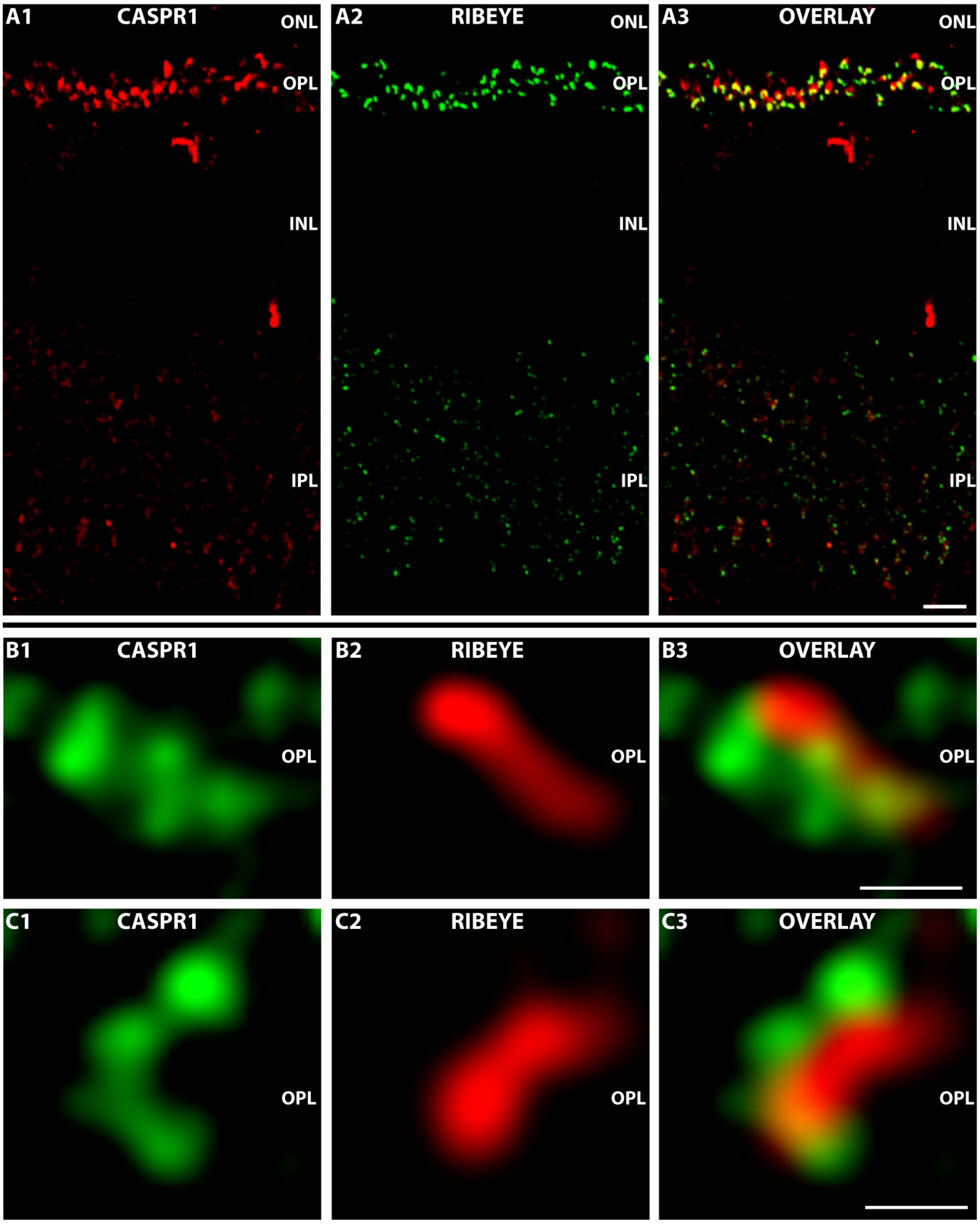

Figure legend: Appendix Figure S6

Semi-thin sections of the mouse retina double-immunolabelled with the mouse monoclonal antibody against CASPR1 and rabbit polyclonal antibody against RIBEYE (U2656; Schmitz et al., 2000). The CASPR1 antibody predominantly labelled the synaptic layers of the retina, the outer plexiform layer (OPL) and the inner plexiform layer (IPL). The synaptic layers were visualized by double-immunolabelling with the antibody against RIBEYE. In the synaptic layers, the CASPR1 immunosignal displayed discrete spot-like distribution enriched in close vicinity to the synaptic ribbon (A). Appendix Fig. S6A was obtained by confocal microscopy. High-resolution SR-SIM microscopy (B,C) of the CASPR1 immunosignals in the OPL where photoreceptor ribbon synapses are located revealed that the CASPR1 immunosignals were highly enriched in close vicinity to the synaptic ribbon. In the incubations in A), B) and C) different secondary antibodies

conjugated to different fluorophores were used on purpose to document that the immunolabelling results are independent from the selected secondary antibody/ fluorophore. ONL, outer nuclear layer; OPL, outer plexiform layer; INL, inner nuclear layer; IPL, inner plexiform layer. Scale bars: 20μm (A); 1μm (B,C).

Appendix Figure S7

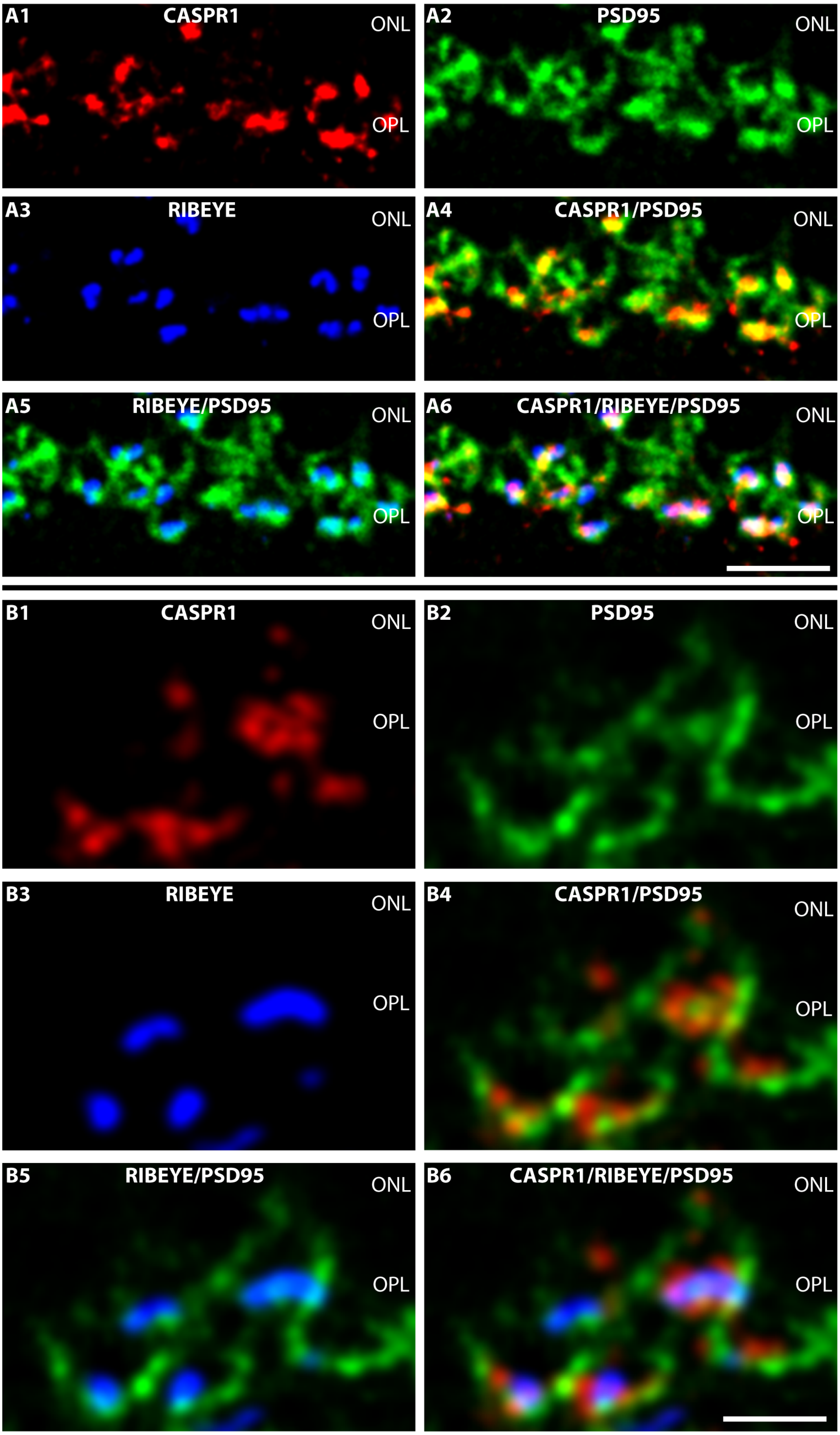

Figure legend: Appendix Figure S7

Confocal images of semi-thin sections of the mouse retina triple-immunolabelled with mouse monoclonal antibodies against CASPR1, rabbit polyclonal antibodies against PSD-95 (L667) and rabbit polyclonal antibodies against RIBEYE (U2656), as described in the Materials and Methods section. In A4,A4 and B4,B5 the indicated two immunosignals are overlayered on each other; in A6, B6, all three immunosignals were overlayered on each other. ONL, outer nuclear layer; OPL, outer plexiform layer. Scale bars: 2μm.

Appendix Figure S8

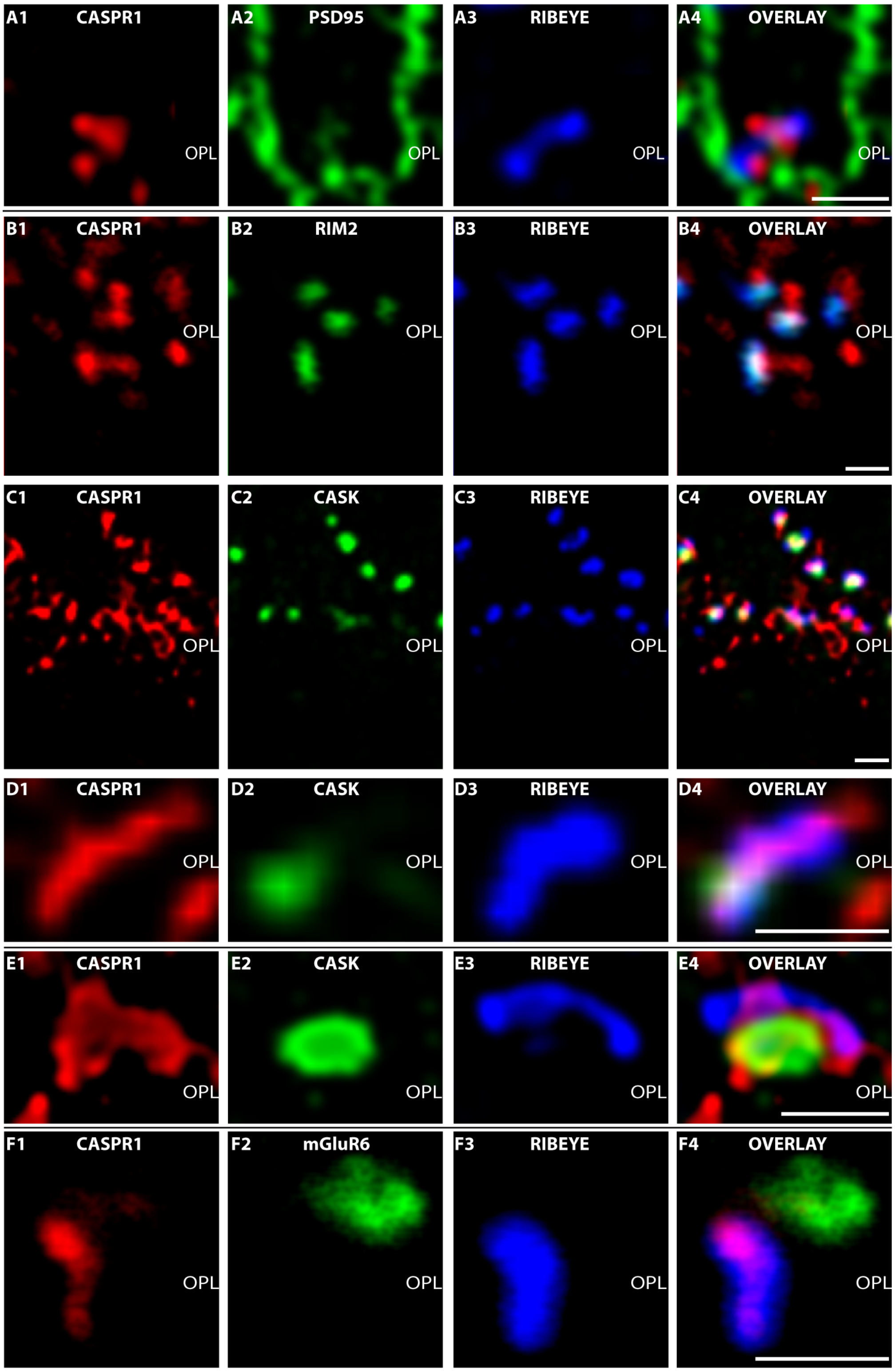

Figure legend: Appendix Figure S8

CASPR1 is located pre-synaptically in close vicinity to the synaptic ribbon. A) High-resolution confocal analysis of rod photoreceptor synapses in the OPL of the mouse retina (0.5μm-thin sections) that were triple-immunolabelled with the rabbit polyclonal antibody against CASPR1, mouse monoclonal antibody 2D9 against RIBEYE and the indicated third primary antibodies (A-F). In (A), the outline of a single presynaptic terminal was visualized by immunolabelling with antibodies against PSD95 (A2). The

CASPR1 immunosignal is located within the presynaptic terminal in close vicinity to the synaptic ribbon. Furthermore, presynaptic CASPR1 was found in close vicinity to the active zone markers RIM2 (B) and CASK (C,D,E). In contrast, the CASPR1 signal did not overlap with the postsynaptic signal for mGluR6 that is localized at the tip of invaginating ON-bipolar cells that are located also in close vicinity to the synaptic ribbon (Appendix Fig. S8F). Appendix Figs. S8B,C,D,F were obtained by confocal microscopy; Appendix Figs. S8A,E by super-resolution structured-illumination-microscopy (SR-SIM). OPL, outer plexiform layer; INL, inner nuclear layer; IPL, inner plexiform layer. Scale bars: 1μm (A-F).

Appendix Figure S9

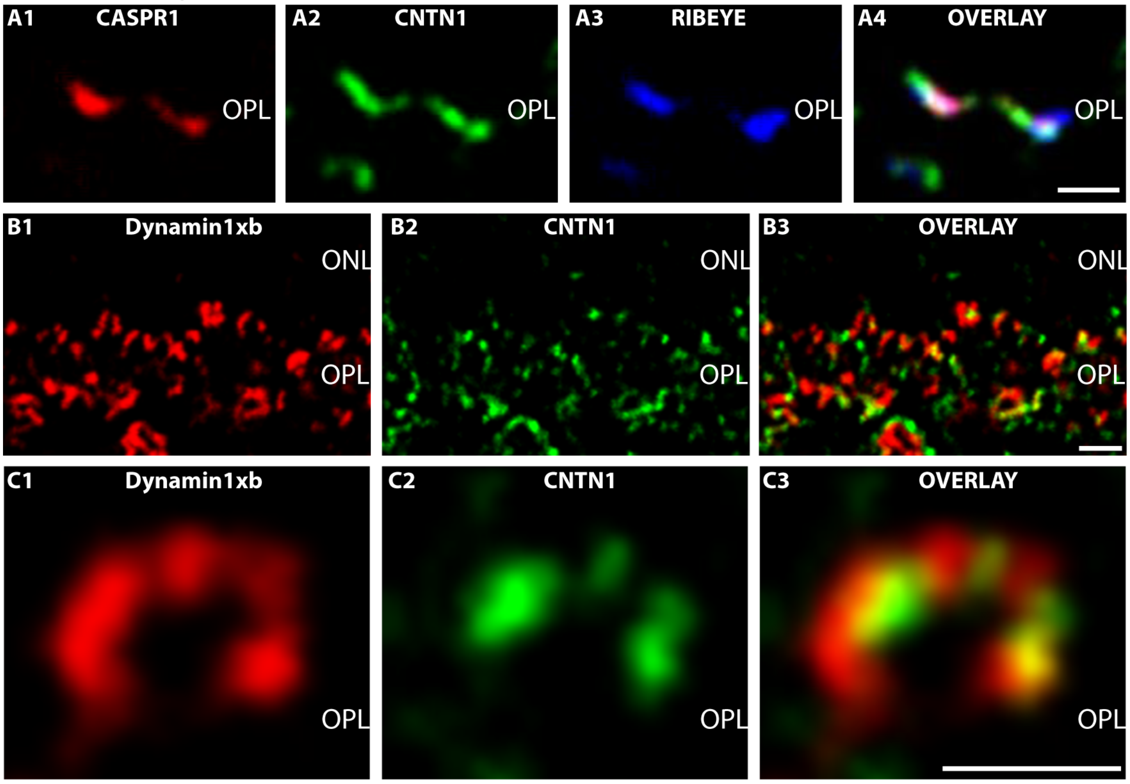

Figure legend: Appendix Figure S9

Contactin-1 (CNTN1) co-localizes with CASPR1 at photoreceptor synapses in the OPL in close vicinity to the synaptic ribbon. (A) High-resolution confocal analyses of rod photoreceptor synapses of the mouse retina (0.5µm-thin sections) triple-immunolabelled with rabbit polyclonal antibody against CASPR1, mouse monoclonal antibody against CNTN1 and rabbit polyclonal antibodies against RIBEYE (U2656). CNTN1 largely has a similar distribution as CASPR1 in photoreceptor synapses of the OPL and is highly enriched at the synaptic ribbon. (B,C) High-resolution confocal analysis of semi-thin (0.5µm-thin) sections of the mouse retina double-immunolabelled with the rabbit polyclonal antibody against CNTN1 and mouse monoclonal antibody against dynamin1xb (clone 1E10; Eich et al., 2017). CNTN1 has a similar distribution as dynamin1xb which is enriched in the presynaptic, peri-active zone in rod photoreceptor synapses (Wahl et al., 2016; Eich et al., 2017). ONL, outer nuclear layer; OPL, outer plexiform layer. Scale bars: 1µm.

Appendix Figure S10

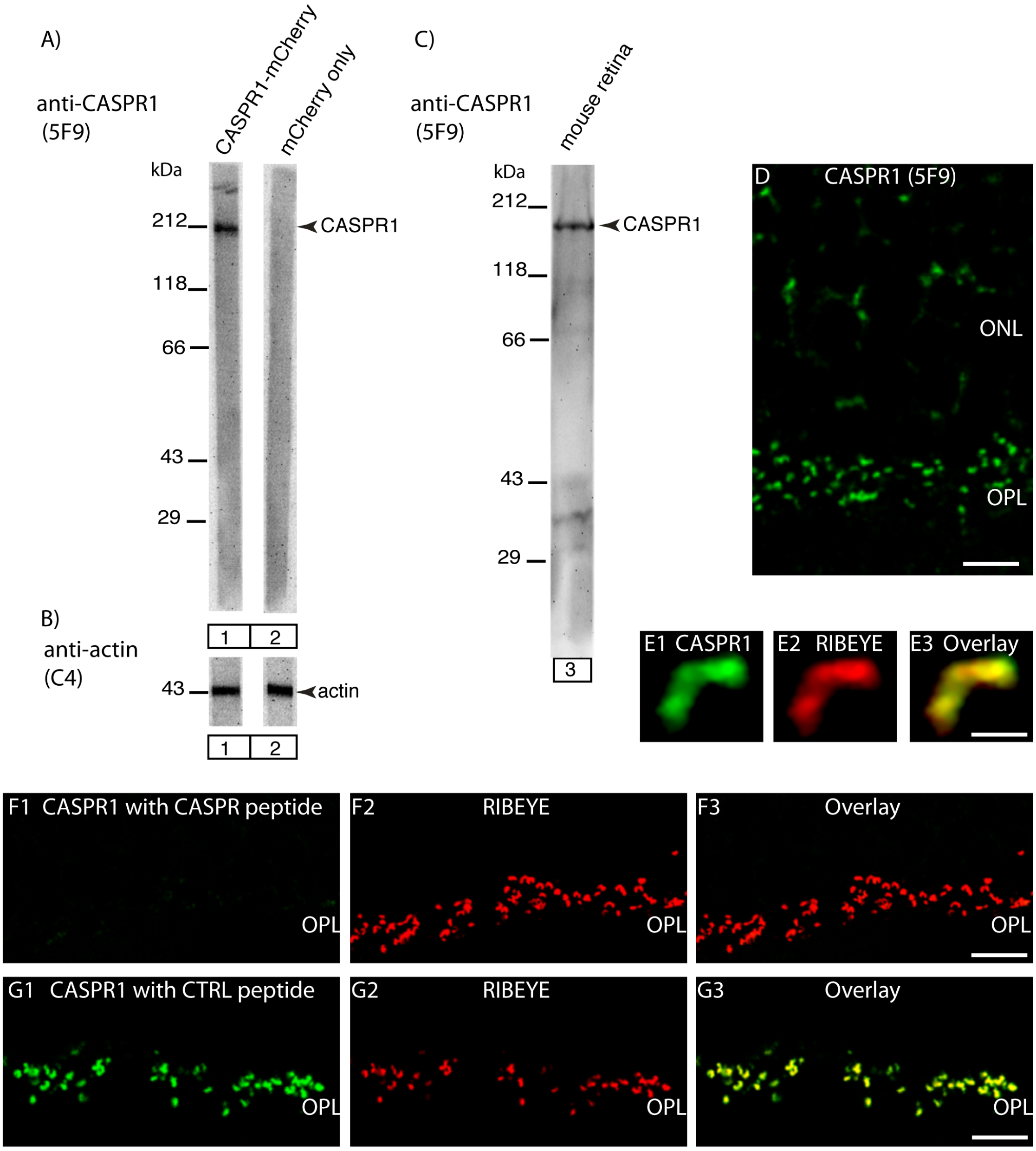

Figure legend: Appendix Figure S10

(A) Western blot analyses of HEK cell extracts that were transfected with mCherry cDNA or CASPR1 mCherry cDNA with the monoclonal anti-CASPR1 antibody (5F9). 5F9 detects CASPR1 only in the CASPR1m-Cherry-transfected COS cells but not in the mCherry-transfected COS cells. A single band at the expected running position was observed. In (B), the same nitrocellulose membranes were re-probed with anti-actin antibodies (C4, 1:2,000 dilution) as loading control. In C), mouse retina was probed with the 5F9 anti-CASPR1 monoclonal antibody in Western blot. The typical protein at ≈190kDa was detected as a single band. (D) 5F9 monoclonal antibody against CASPR1 produced a strong synaptic staining in the OPL. (E) High-resolution immunolabelling of a single synaptic ribbon with monoclonal anti-CASPR1 (5F9, E1) and polyclonal anti-RIBEYE antibody (E2). Overlay image is shown in (E3). This synaptic staining was blocked by pre-absorption with the CASPR1 peptide (F1) but not by pre-absorption with a control peptide (G1). F2,G2 represents an independent reference immunolabelling (anti-RIBEYE); F3,G3 are the corresponding overlay images. ONL, outer nuclear layer; OPL, outer plexiform layer. Scale bars: 5μm.

Appendix Figure S11

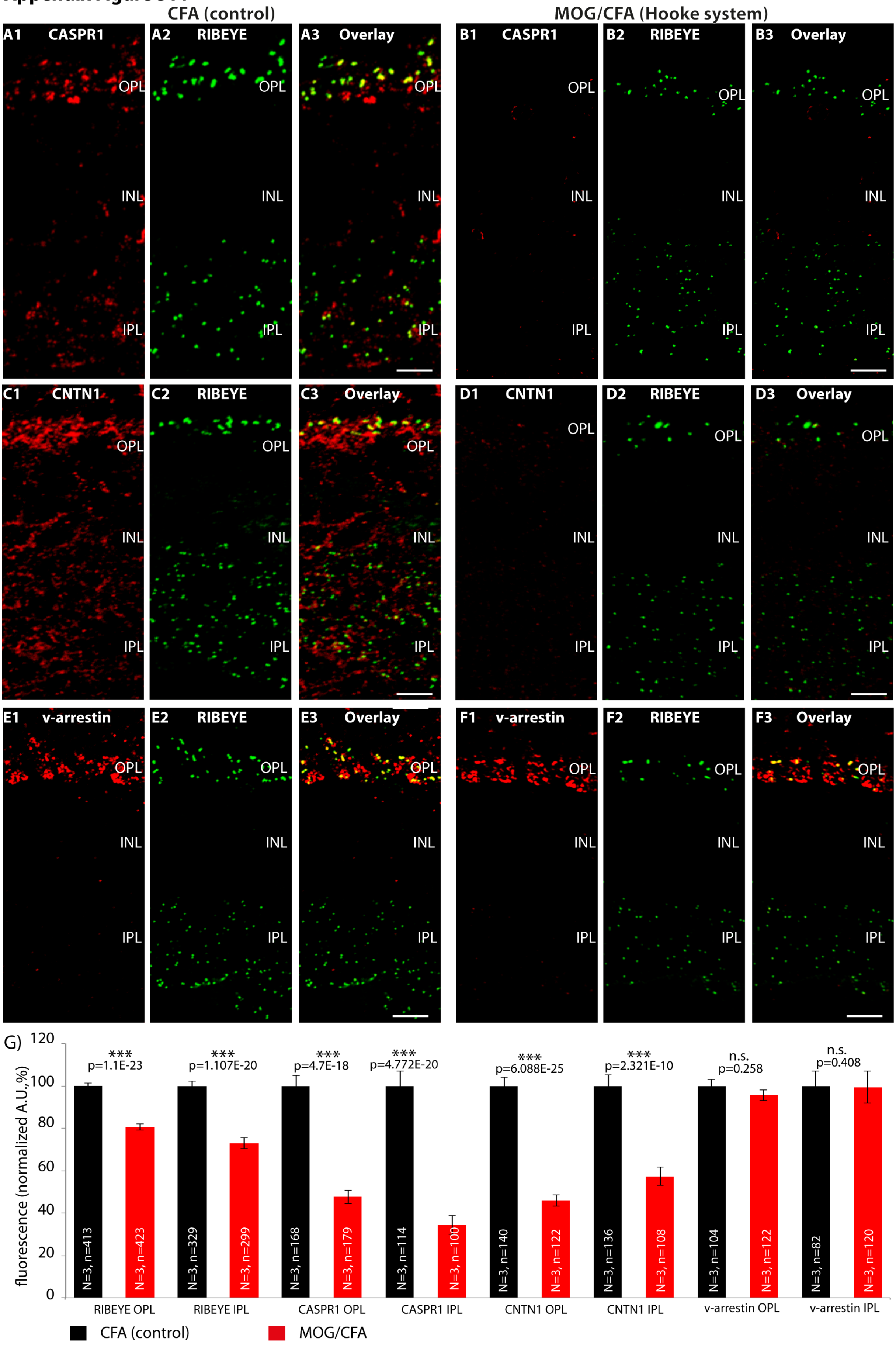

Figure legend: Appendix Figure S11

Localization of CASPR1/CNTN1/vis-arrestin and RIBEYE in semi-thin sections of retinas from either MOG/CFA-injected mice (EAE mice) or CFA-injected control mice (9 days after injection). Mice were injected with commercial suspensions (Hooke kit). Semi-thin sections were double-immunolabelled with antibodies against CASPR1 and RIBEYE (A-B), or with antibodies against v-arrestin and RIBEYE (C-D), or with antibodies against CNTN1 and RIBEYE (E-F). Note that for convenience of comparison the images of the immunolabelled retinas from MOG/CFA-injected animals vs. CFA-injected control animals were displayed in a mirror-imaged manner. Immunosignals were quantified in G. N=3 pairs of mice. RIBEYE quantification: n=413 slices (for OPL analysis (CFA); n=423 slices for MOG; RIBEYE quantification: n=329 slices for IPL analysis (CFA) and 299 slices for MOG; CASPR1 quantification: n=168 slices for OPL analysis (CFA), n=179 slices (for MOG); n=134 slices for IPL (CFA), n=100 slices for MOG; CNTN1 quantifications: n=140 slices for OPL analyses (CFA), n=122 slices for MOG; n=130 slices for IPL analyses, n=108 for MOG; visual arrestin quantification: n=104 slices for OPL analyses (CFA), n=122 slices for MOG; n=82 slices for IPL analyses (IPL), n=120 for MOG. Abbreviations: v-arrestin, visual arrestin; OPL, outer plexiform layer; INL, nuclear layer; IPL, inner plexiform layer. Scale bars: 20μm.

Appendix Figure S12

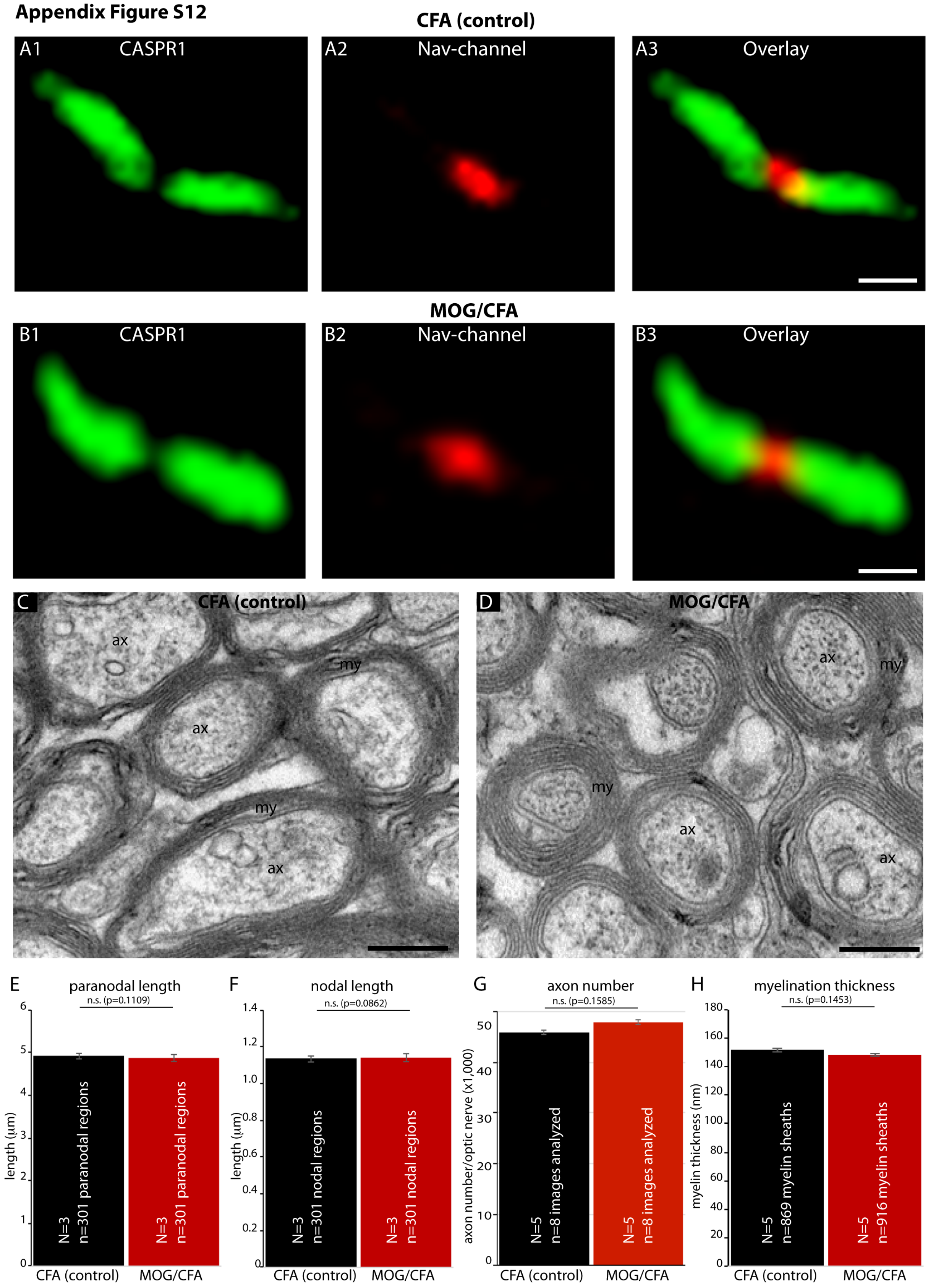

Figure legend: Appendix Figure S12

Analyses of optic nerve samples from mice that were injected with the commercially available MOG/CFA suspensions (Hooke kit; for comparison with own lab-made MOG/CFA suspensions, see Fig. 5). (A,B) Cryostat sections of the optic nerve from EAE and control mice were stained with anti-Na_v antibody to label the node of

Ranvier and with anti-CASPR1 antibody to stain the paranodal region. Length of the node of Ranvier and length of the paranodal region were quantified in E) and F). (C,D) transmission electron micrographs of the optic nerves from MOG/CFA- and CFA-injected animals. Axon numbers and myelin thickness were quantified in G) H). Abbreviations: ax, axon; my, myelin sheath. Scale bars: 2μm (A,B); 1μm (C,D).

Appendix Figure S13

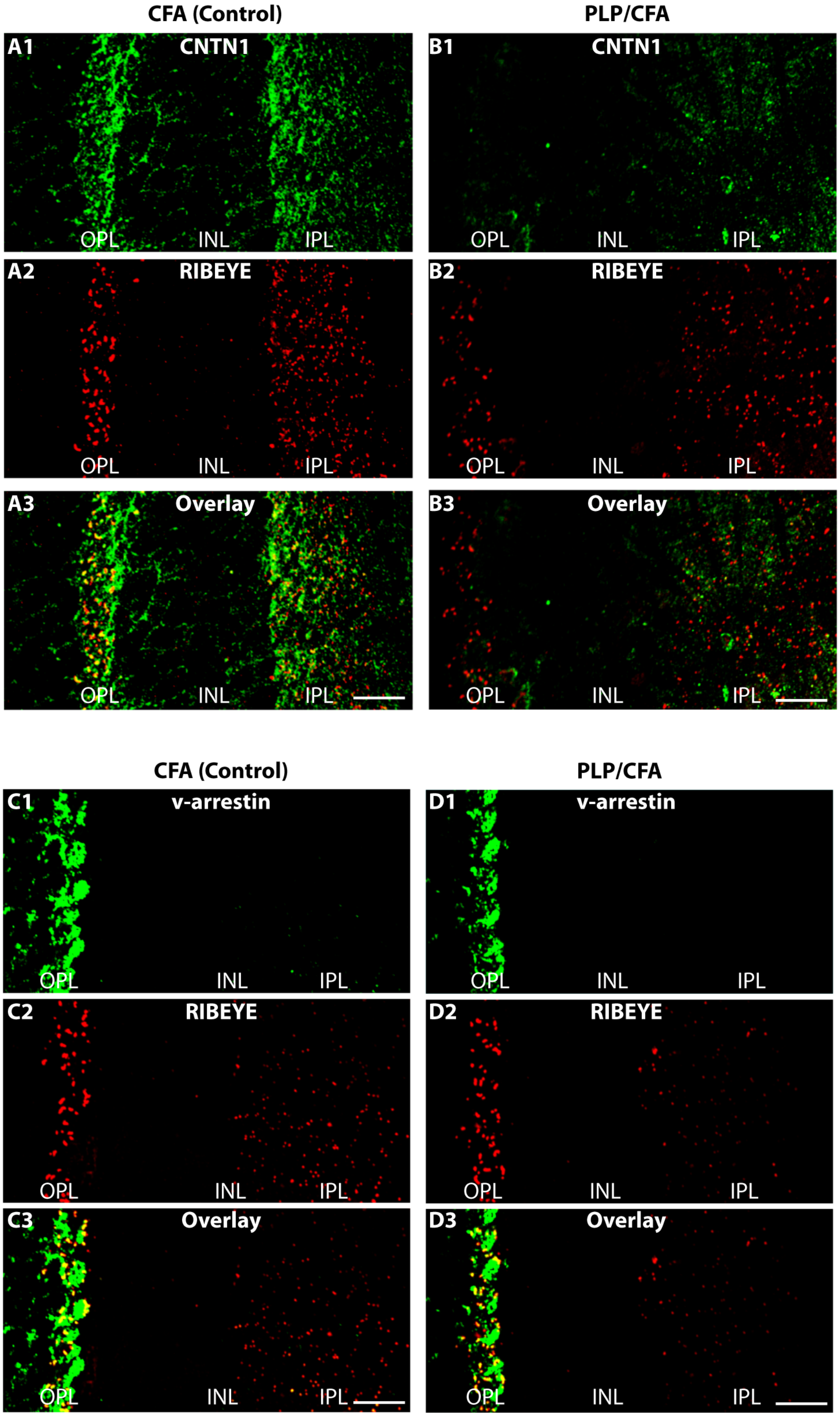

Figure legend: Appendix Figure S13

Localization of CASPR1 and visual-arrestin (v-arrestin) in semi-thin sections of retinas from either PLP/CFA-injected mice (EAE mice) or CFA-injected control mice (9 days after injection). Semi-thin sections were double-immunolabelled with antibodies against CNTN1 and RIBEYE (A-B) or with antibodies against v-arrestin and RIBEYE (C-D). For quantification of the immunolabelling data, please see Fig. 7C. v-arrestin, visual arrestin; OPL, outer plexiform layer; INL, nuclear layer; IPL, inner plexiform layer. Scale bars: 20µm.

Appendix Figure S14

A)

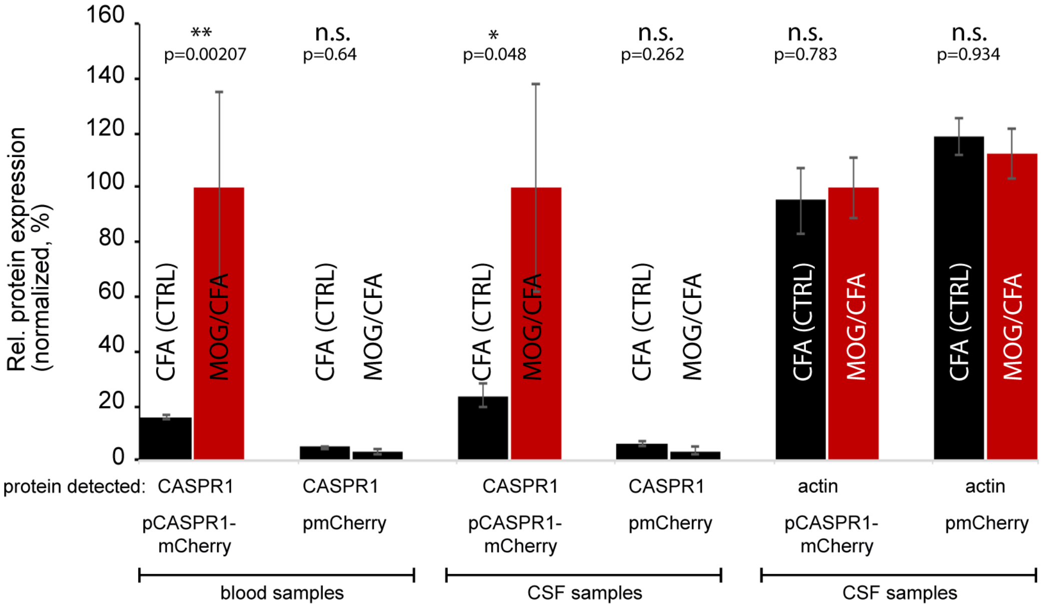

B)

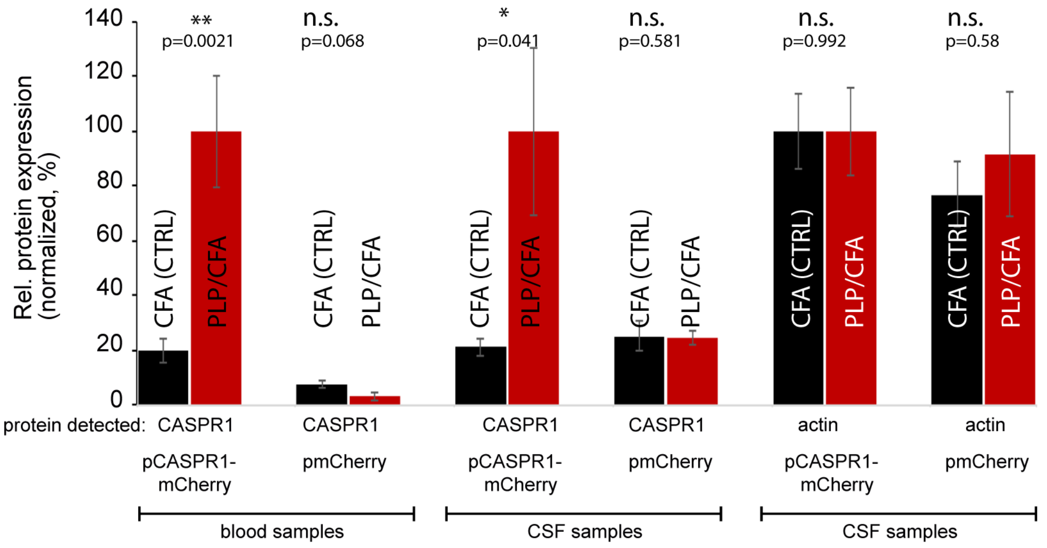

Figure legend: Appendix Figure S14

Quantification of the Western blot data from Figure EV2 shown as mean ± S.E.M. N=3 pairs of mice for MOG/CFA vs. CFA-injected control mice (commercial system; Hooke kit; A) and 6 pairs of mice for PLP/CFA- vs. CFA-injected control mice (in B). **, p<0.01; *, p<0.05; n.s., non-significant (unpaired two-tailed Student's t-test); precise p-values are given in the figure.

Appendix Figure S15

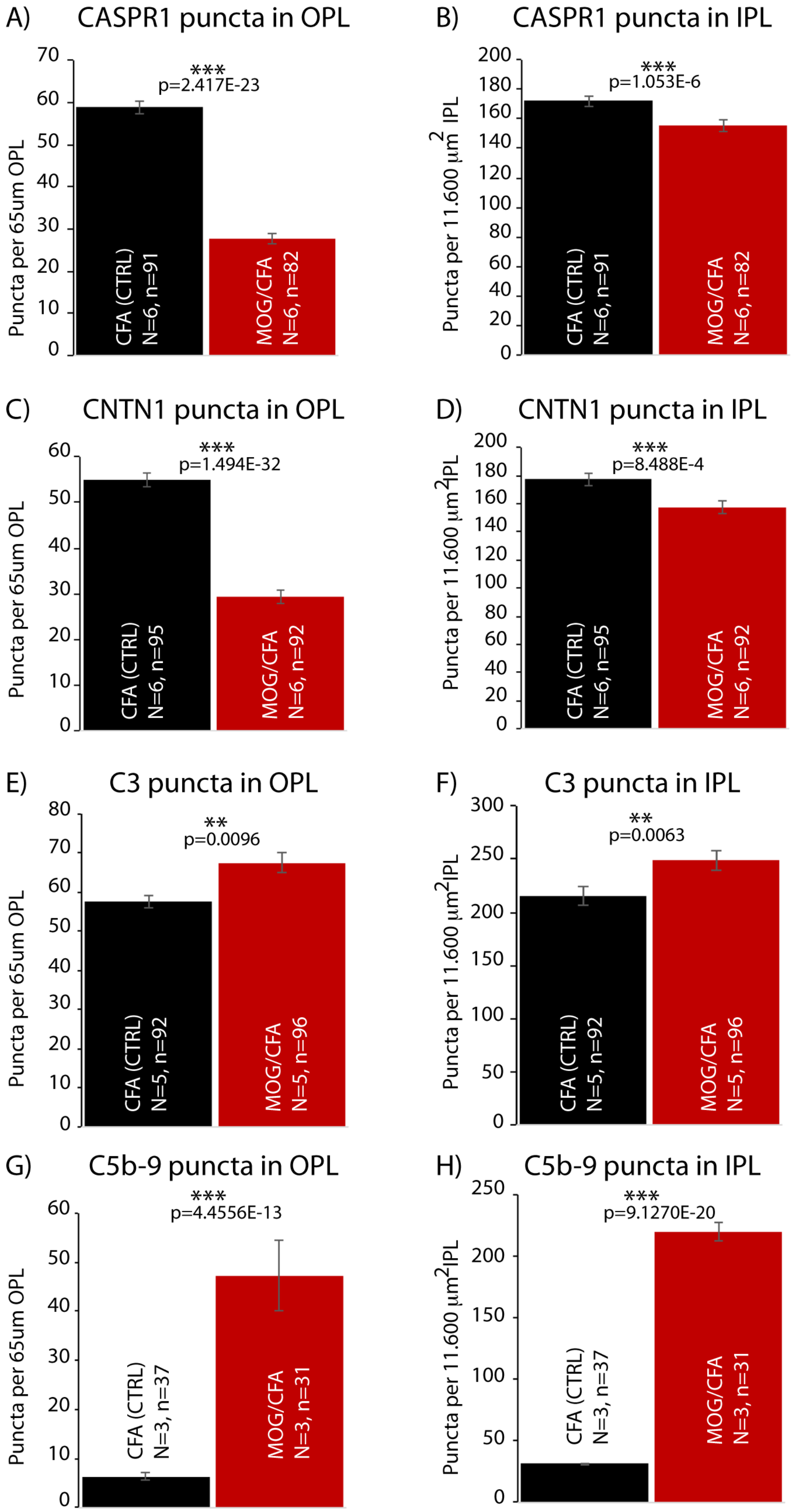

Figure legend: Appendix Figure S15

(A-E) Quantification of CASPR, CNTN1 and C3 puncta in OPL and IPL from MOF/CFA and CFA-injected control mice. Quantification was performed from the experiments demonstrated in Figs 2,3; Figs EV3 and EV4. F) Quantification of C5b-9 immunofluorescence signals (integrated density) from Fig. EV4.
