# Supplementary material for: Early auto‐immune targeting of photoreceptor ribbon synapses in mouse models of multiple sclerosis
Source: EMBO Mol Med. 2018 Sep 28;10(11):e8926. doi: 10.15252/emmm.201808926 (PMC6220320; doi:10.15252/emmm.201808926)
Supplement: Supplementary file 2 — Expanded View Figures PDF [file EMMM-10-e8926-s002.pdf]

Expanded View Figures

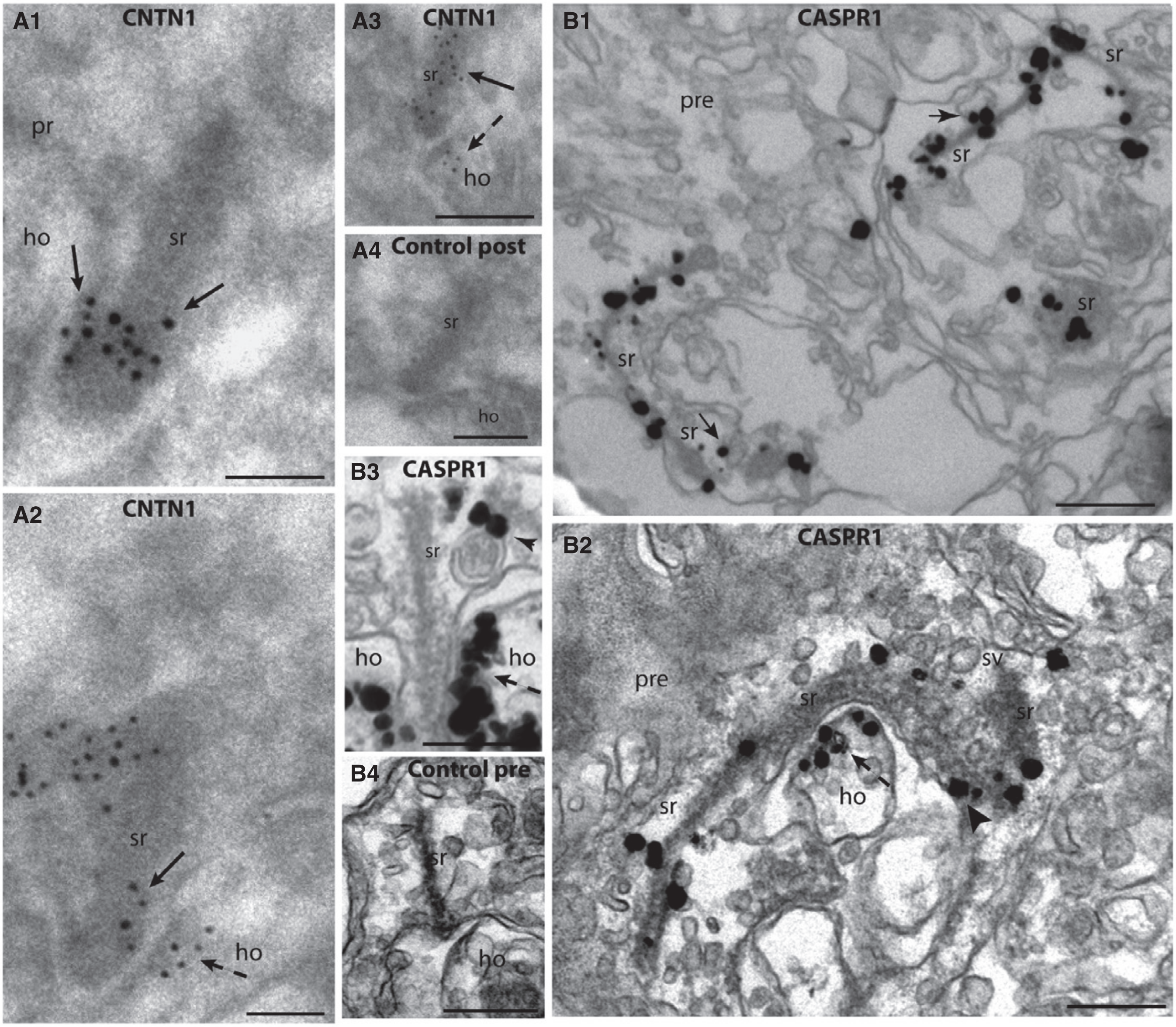

Figure EV1. Ultrastructural localization of CASPR1 and CNTN1 in photoreceptor ribbon synapses.

A, B Photoreceptor ribbon synapses were analyzed because of their clear pre- and postsynaptic organization. The ultrastructural localization of CNTN1 was obtained by post-embedding immunogold electron microscopy (A1–A3; A4 is a negative control). The ultrastructural localization of CASPR1 was obtained by pre-embedding electron microscopy using flash-frozen, unfixed cryostat section of the mouse retina (B1–B3; B4 is a negative control). Both CNTN1 and CASPR1 were found to be localized at the synaptic ribbon. Cross-sections of the ribbon are demonstrated in (A1–A4, B3, B4); (B1 and B2) show tangential sections of the ribbon. Endo-membranes are only weakly visible in post-embedding microscopy because tissue cannot be osmicated. Pre-embedding immunogold microscopy indicates that CASPR1 is found on vesicles that are attached to the synaptic ribbon (arrows in B1). In addition to the labeling of the synaptic ribbon complex, we also found immunolabeling of the presynaptic plasma membrane of the active zone (arrows in A1, A2) and of the postsynaptic plasma membrane of horizontal cells in direct apposition to the active zone (dashed arrows in A2, B2, B3). Arrowhead in (B3) shows CASPR1 immunosignal on a vesicle in the peri-active zone, a site of endocytic vesicle retrieval (Wahl *et al*, 2013, 2016). The arrowhead in (B2) denotes a presynaptic plasma membrane-associated CASPR1 immunosignal. pr, presynaptic; ho, postsynaptic tip of a horizontal cell; sr, synaptic ribbon; pre, presynaptic terminal; sv, synaptic vesicle; ho, horizontal cell. Scale bars: 150 nm (A1), 100 nm (A2), 200 nm (A3), 150 nm (A4), 250 nm (B1, B2), 150 nm (B3), 100 nm (B4).

© 2018 The Authors

Figure EV2. Serum and cerebrospinal fluid from MOG/CFA- or PLP/CFA-injected mice contain auto-reactive antibodies against CASPR1.

A–F The indicated serum and cerebrospinal fluid (CSF) samples obtained from MOG/CFA-injected, CFA-injected (A–C; injected with commercial MOG/CFA suspensions; Hooke kit) or PLP/CFA- and CFA-injected mice (D–F) were probed for their reactivity with CASPR1. CASPR1 was heterologously expressed in HEK293 cells as CASPR1-mCherry fusion protein; mCherry alone served as control protein. Serum was collected from the indicated mice before and after injection. We observed a strong reactivity of both blood samples as well as CSF samples with CASPR1-mCherry but not mCherry alone. Only post-injection samples from MOG/CFA- or PLP/CFA-injected mice (lanes A3, A4, D3, F3), but not pre-injection samples (C1–C4, D1, F1), showed a strong reactivity against CASPR1. Neither post- nor pre-injection samples from control-injected mice (A1, A2, B1–B4, C3, E1, E3) showed a reactivity against CASPR1. For quantification, see Appendix Fig S14. N = 3 pairs of mice (MOG/CFA; commercial suspensions); N = 6 pairs of mice (PLP/CFA suspensions). Please note that blots shown in (A and D) were re-probed (without stripping of the blot) with anti-actin antibody (as loading reference).

© 2018 The Authors

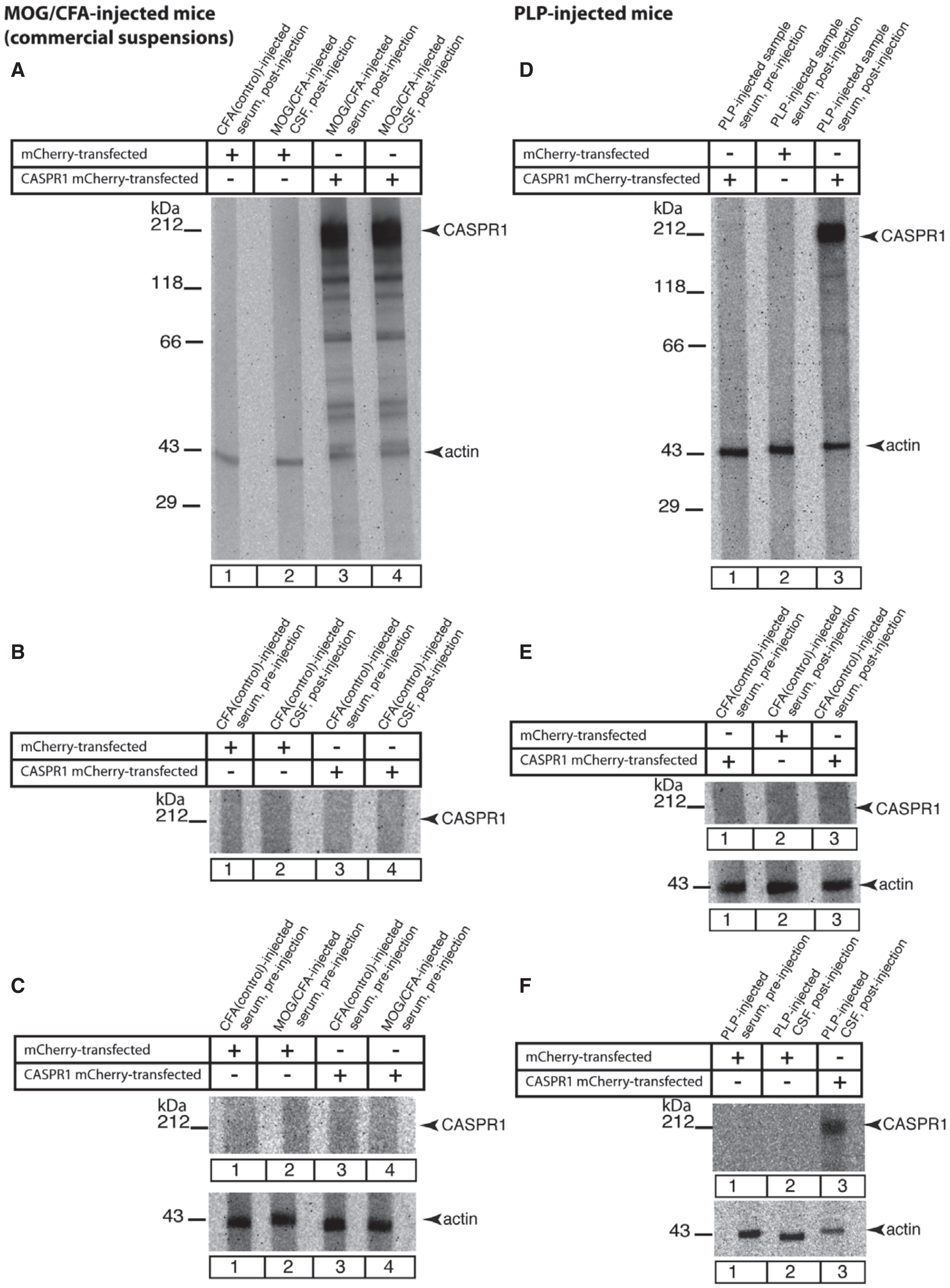
MOG/CFA-injected mice
(commercial suspensions)
A
CFA(control)-injected serum, post-injection
MOG/CFA-injected CSF, post-injection
MOG/CFA-injected serum, post-injection
MOG/CFA-injected CSF, post-injection
mCherry-transfected
CASPR1 mCherry-transfected
kDa
212
118
66
43
29
CASPR1
actin
B
CFA(control)-injected serum, pre-injection
CFA(control)-injected CSF, post-injection
C
MOG/CFA-injected serum, pre-injection
PLP-injected mice
D
PLP-injected sample serum, pre-injection
PLP-injected sample serum, post-injection
E
CFA(control)-injected serum, post-injection
F
PLP-injected serum, pre-injection
PLP-injected CSF, post-injection

Figure EV2.

Figure EV3. Increased recruitment of complement protein C3 to retinal synapses after MOG/CFA injection.

A, B Semi-thin (0.5-μm-thin) sections of the mouse retina from MOG/CFA-injected mice (injected with self-made MOG/CFA suspensions) in comparison with CFA-injected control mice double-immunolabeled with rabbit polyclonal antibody against complement protein 3 (C3) and mouse monoclonal antibody against RIBEYE (2D9). Scale bars: 20 μm.

C Quantification of C3 immunosignals in the synaptic layers (integrated immunofluorescence staining intensities). $N = 5$ independent experiments; $n = 92$ sections (CFA); $n = 96$ sections (MOG). ONL, outer nuclear layer; OPL, outer plexiform layer; INL, inner nuclear layer; IPL, inner plexiform layer; GCL, ganglion cell layer. ***$P < 0.001$ (precise P-values are given in the figure).

D Full-length complement C3 protein (at ≈180 kDa) was detected by Western blot in retinas from both MOG/CFA-injected mice and CFA-injected control mice (as well as in non-injected C57Bl/6 mice; data not shown). In MOG/CFA-injected samples, we frequently observed an additional band at ≈120 kDa indicating a possible proteolytic activation of C3 into C3c in retinas of MOG/CFA-injected mice.

E Quantification of Western blot bands shown in (D). $N = 3$ CFA MOG/CFA retina samples.

Data information: Error bars are ± SEM; statistical test: Mann–Whitney U-test (Origin Pro).

© 2018 The Authors

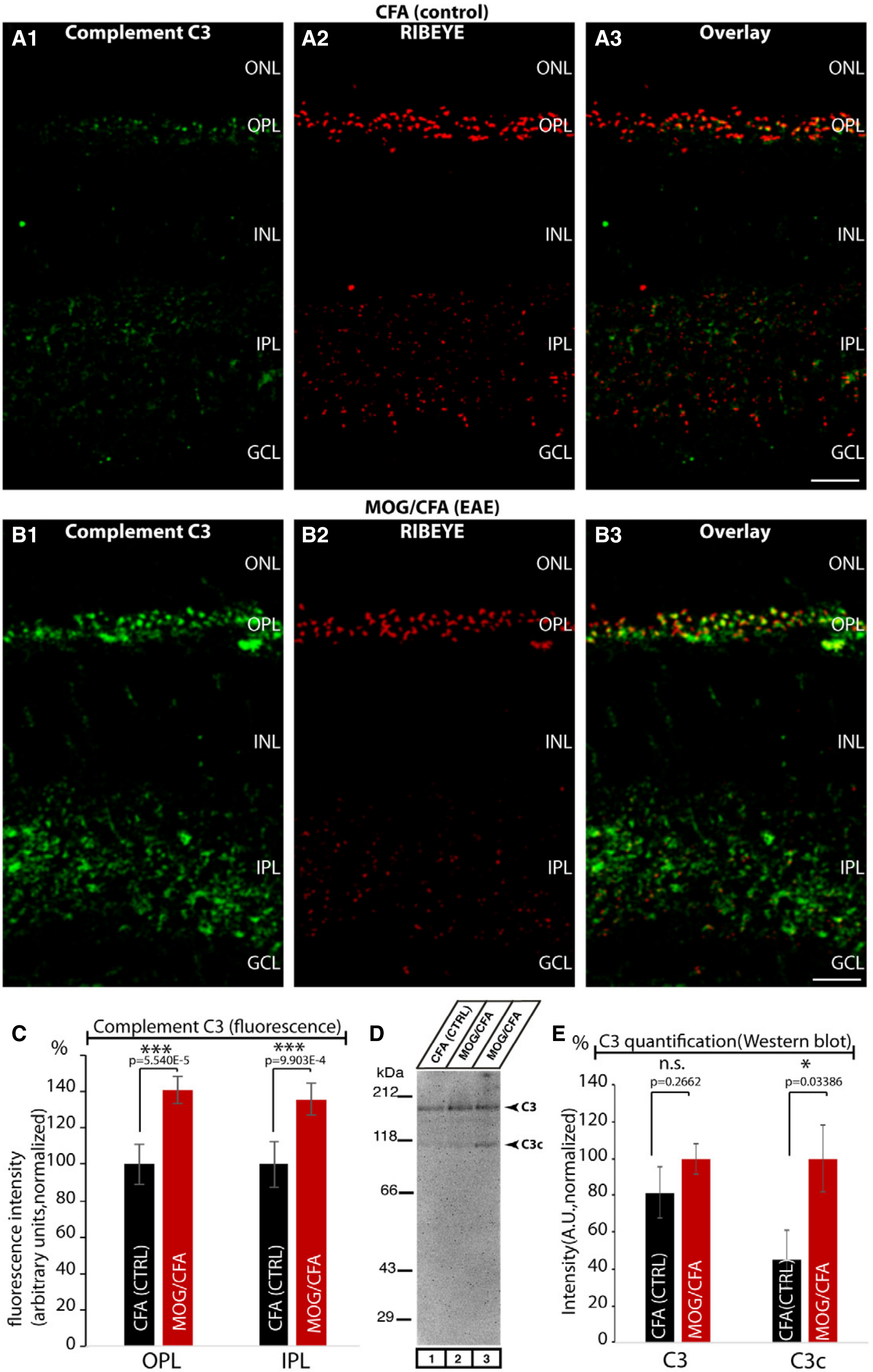
CFA (control)
A1 Complement C3
A2 RIBEYE
A3 Overlay
ONL
OPL
INL
IPL
GCL
MOG/CFA (EAE)
B1 Complement C3
B2 RIBEYE
B3 Overlay
C
Complement C3 (fluorescence)
%
***
p=5.540E-5
***
p=9.903E-4
fluorescence intensity (arbitrary units,normalized)
CFA (CTRL)
MOG/CFA
OPL
IPL
D
CFA (CTRL)
MOG/CFA
MOG/CFA
kDa
212
118
66
43
29
C3
C3c
1 2 3
E
% C3 quantification(Western blot)
n.s.
p=0.2662
*
p=0.03386
Intensity(A.U.,normalized)
C3
C3c

Figure EV3.

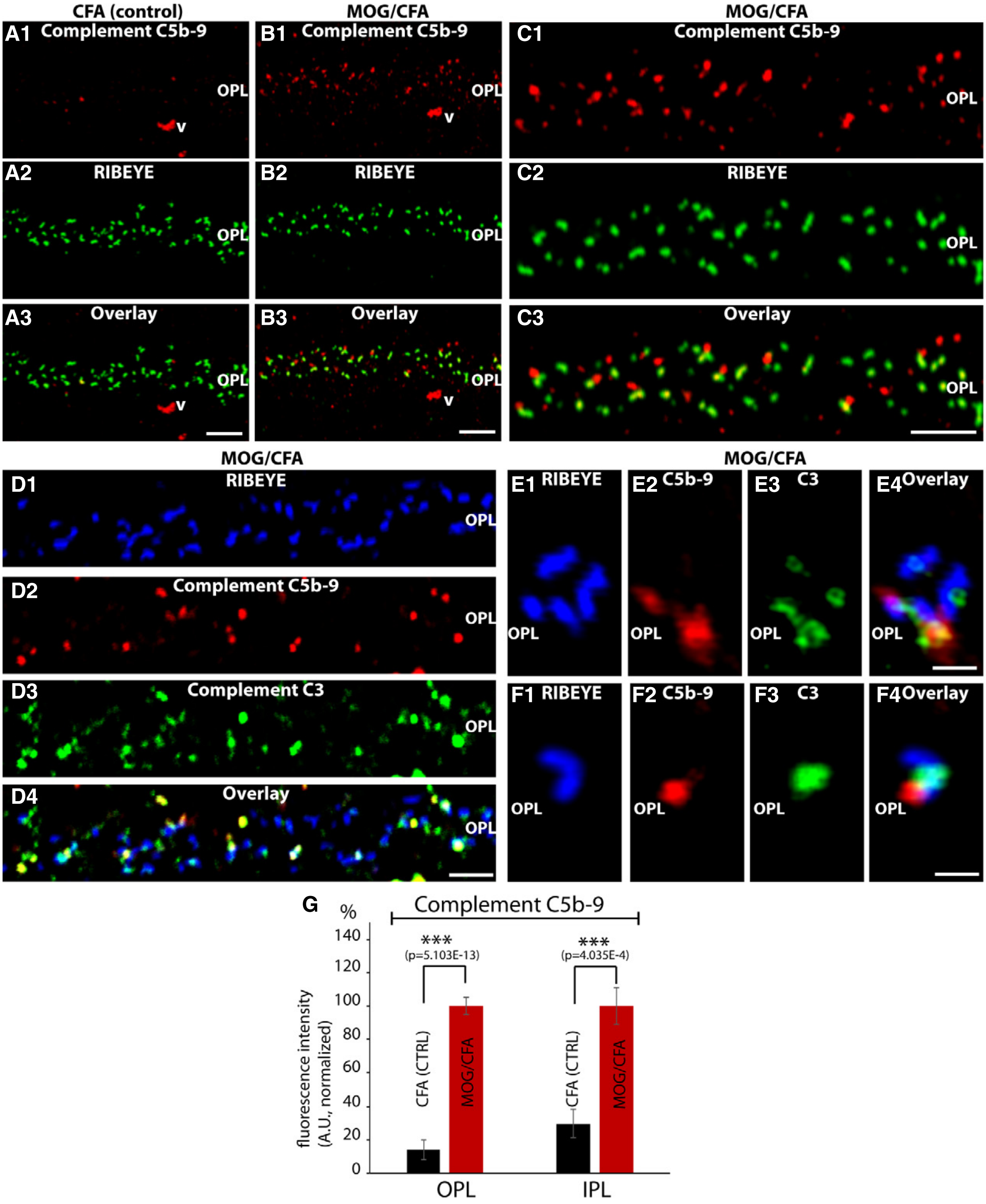

Figure EV4.

    © 2018 The Authors

Figure EV4. Activation of the complement system at retinal synapses after MOG/CFA injection.

A–C Semi-thin (0.5-µm-thin) sections of the mouse retina from MOG/CFA-injected mice (B, C; injected with self-made MOG/CFA suspensions) or CFA-injected control mice (A) double-immunolabeled with the mouse monoclonal antibody C5b-9 that detects a conformational epitope in the assembled, activated terminal complement complex (TCC) and polyclonal antibody against RIBEYE. The C5b-9 neo-epitope is only present in the assembled terminal complement complex (Fluiter *et al*, 2014; Michailidou *et al*, 2015) and can be used to detect the activated TCC. The activated TCC complex is present only in MOG/CFA-injected mice but not in CFA-injected control mice (see also quantification in G).

D–F The activated TCC complex is found close to the synaptic ribbons as visualized by co-immunolabeling with antibodies against RIBEYE. The activated TCC complex, as detected by anti-C5b-9 immunolabeling, co-localized with the immunosignals for complement protein 3 (C3) at photoreceptor synapses from MOG/CFA-injected mice.

G Fluorescence intensity values were normalized to MOG/CFA. OPL, outer plexiform layer; v, vessel; C3, complement protein 3. Quantification of C5b-9 expression in the OPL and IPL of MOG/CFA-injected mice and CFA-injected control mice. $N = 3$; $n = 37$ sections for CFA; $n = 31$ sections for MOG/CFA. ***$P < 0.001$ (precise P-values are given in the figure). Error bars are ± SEM; statistical test: Mann–Whitney U-test (Origin Pro).

Data information: Scale bars: 5 µm (A–C), 3 µm (D), 1 µm (E, F).

© 2018 The Authors

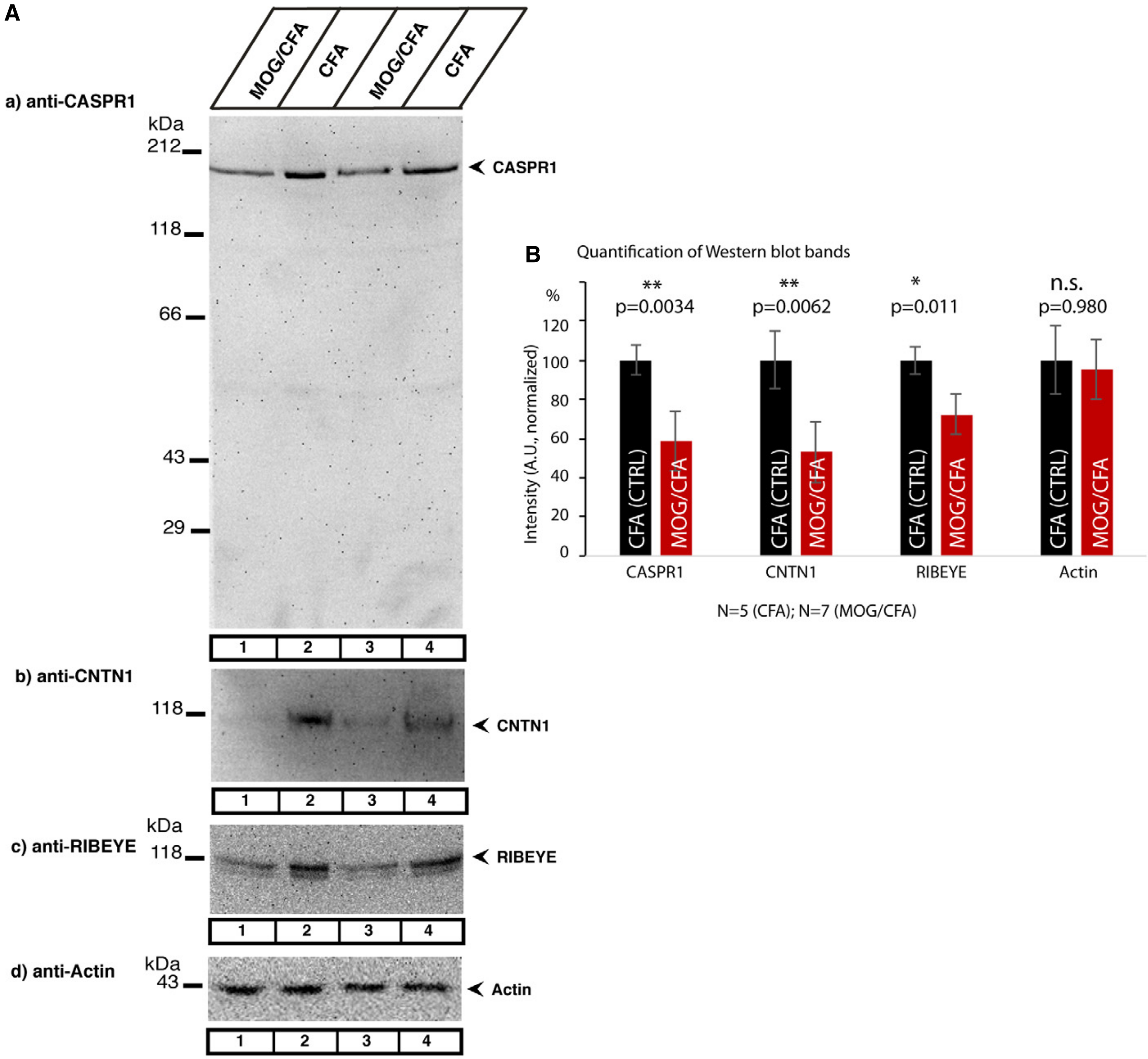

Figure EV5. Retinal CASPR1, CNTN1 and RIBEYE are down-regulated after MOG/CFA injection.

A Retinal lysates from MOG/CFA-injected and CFA-injected control mice tested in Western blot with the indicated antibodies.

B Semi-quantitative evaluation of the band intensity of the proteins recognized in Western blot. n.s., non-significant; $*P < 0.05$; $**P < 0.01$ (precise *P*-values are given in the figure). $N = 5$ retinas from CFA-injected mice; $N = 7$ retinas from MOG/CFA-injected mice. Error bars are ± SEM; statistical test: Mann–Whitney *U*-test (Origin Pro).

© 2018 The Authors
