# Supplementary material for: Early auto‐immune targeting of photoreceptor ribbon synapses in mouse models of multiple sclerosis
Source: EMBO Mol Med. 2018 Sep 28;10(11):e8926. doi: 10.15252/emmm.201808926 (PMC6220320; doi:10.15252/emmm.201808926)

Figure 1A

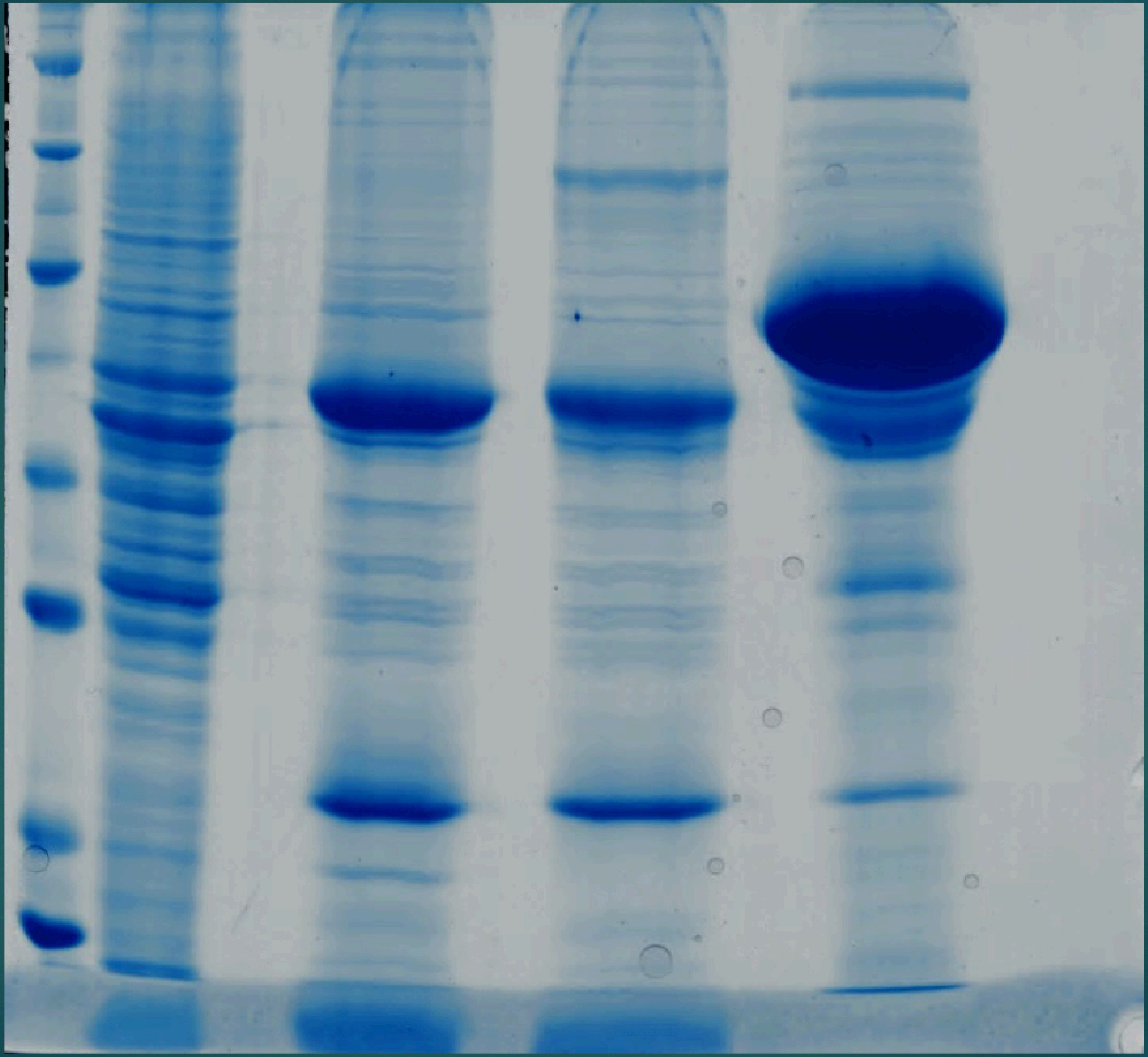

Figure 1Ba

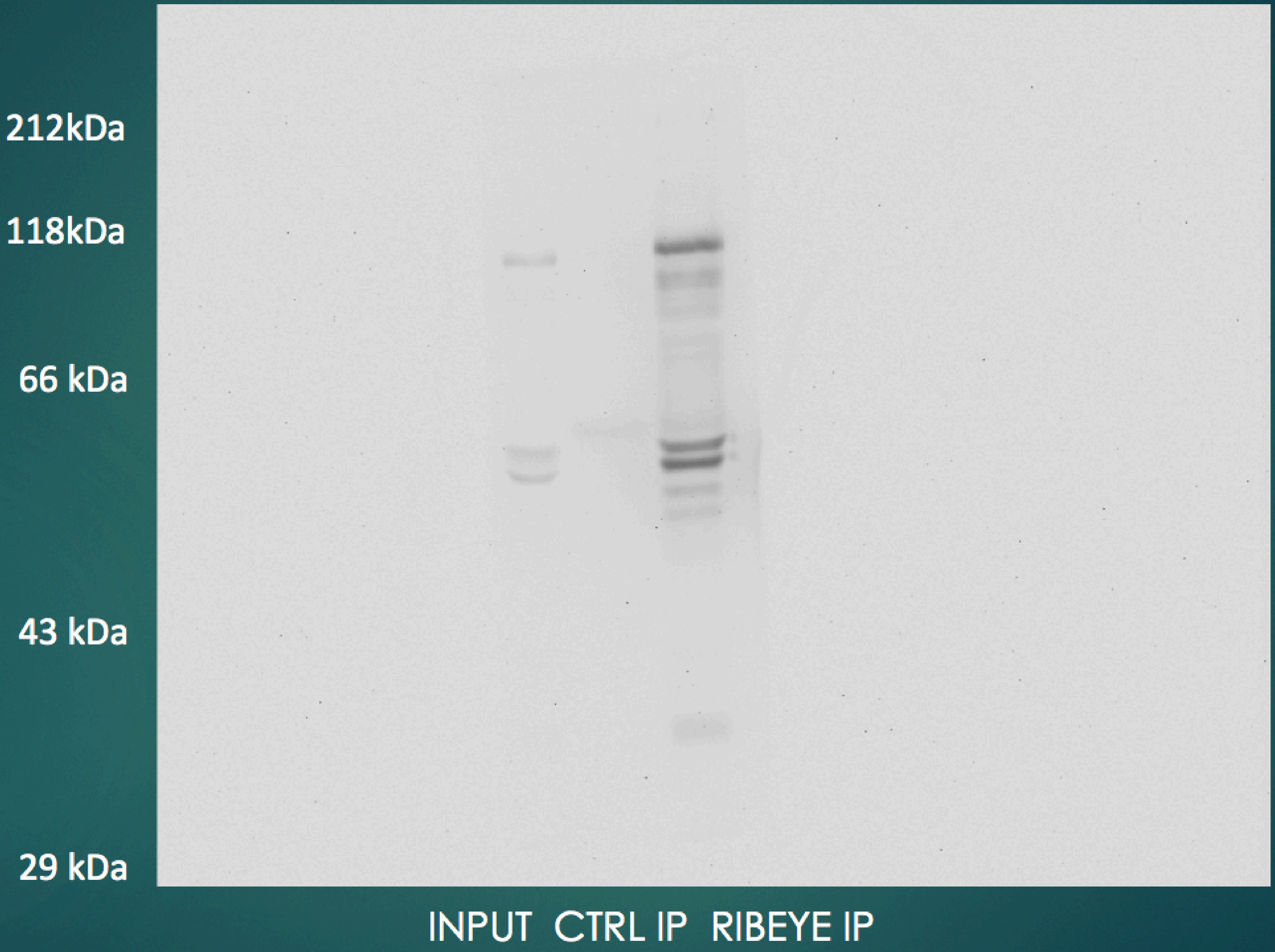

Figure 1Bb

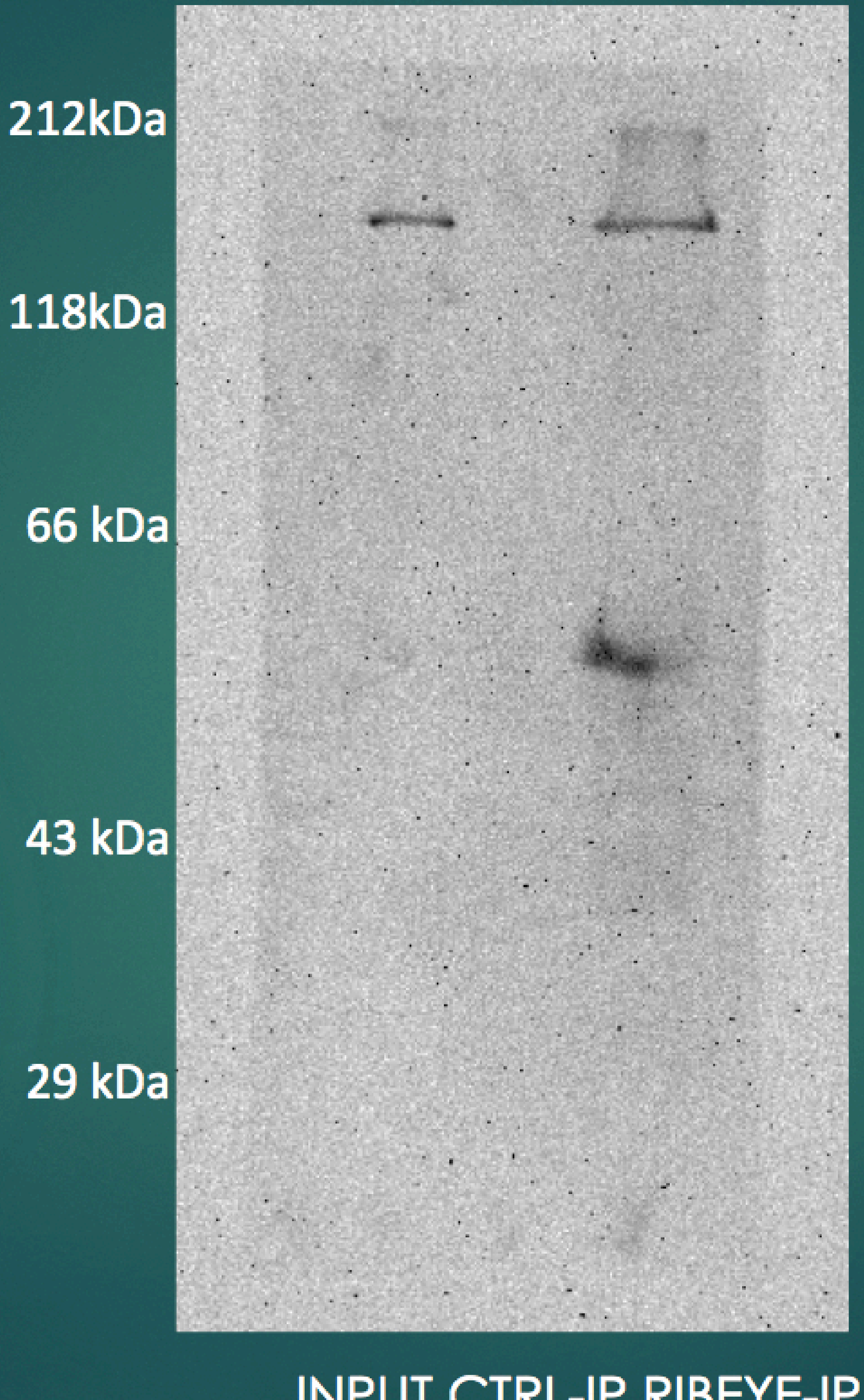

Figure 1Bc

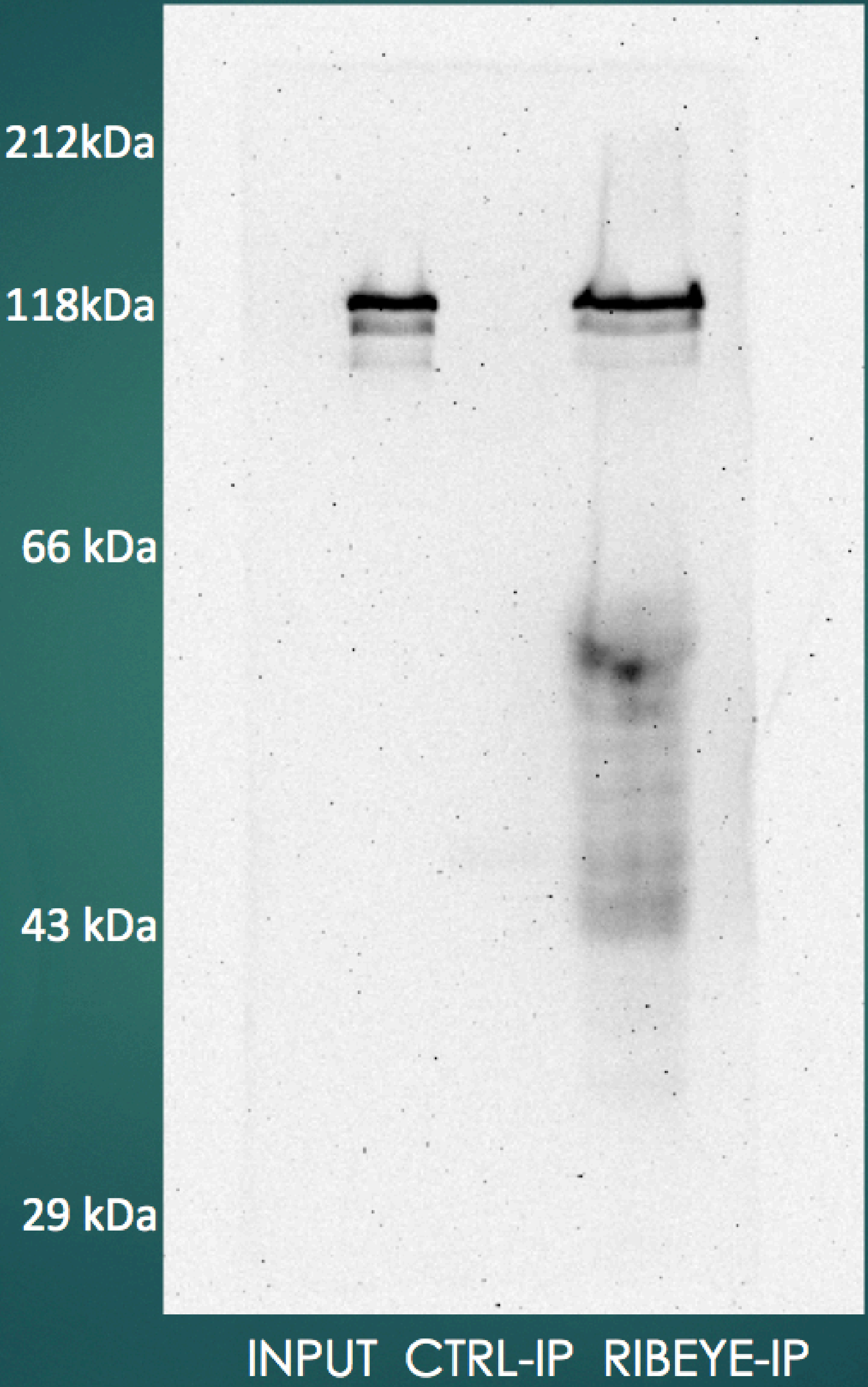

Figure 1Bd

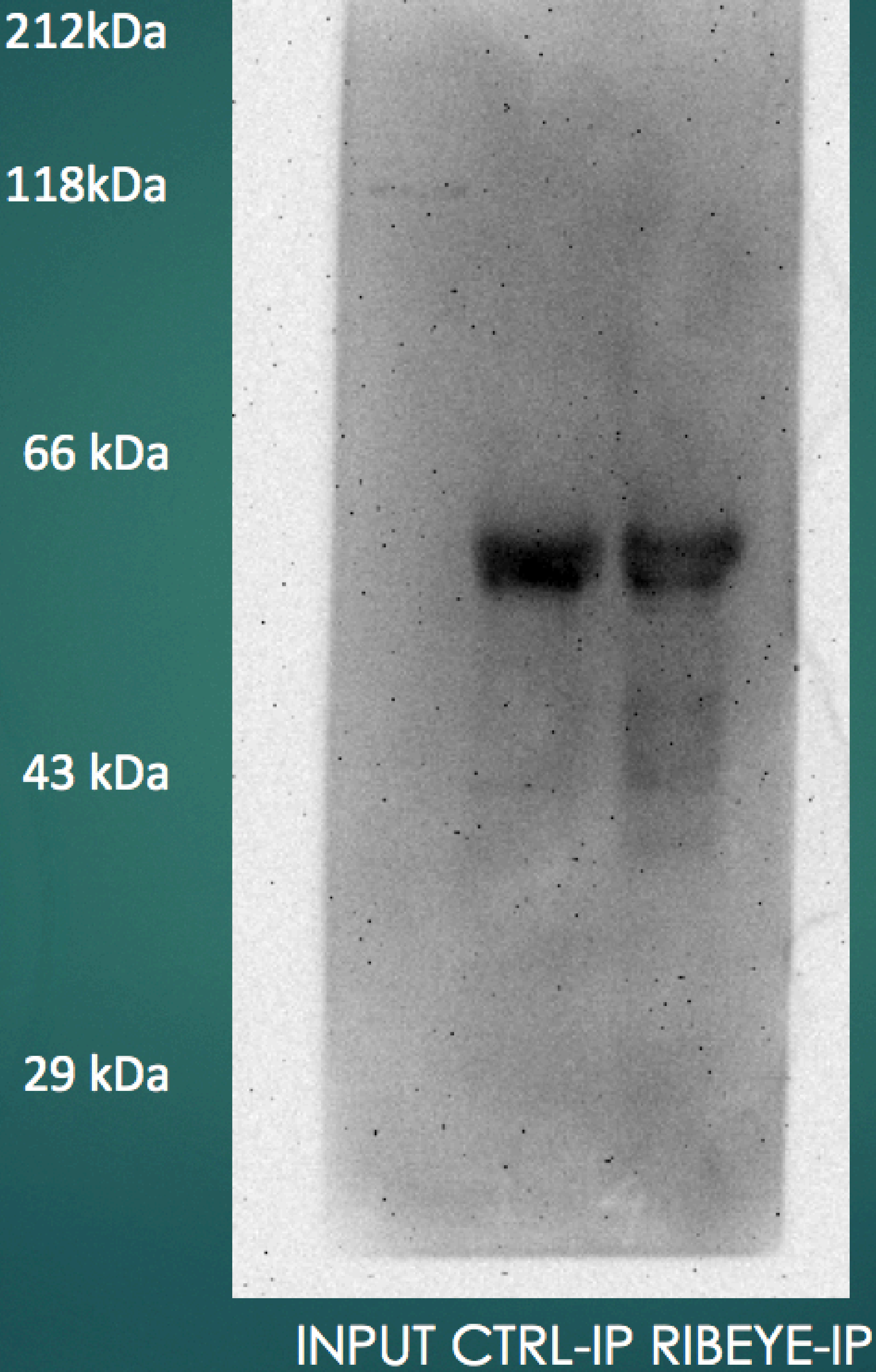

Supplement: Supplementary file 4 — Source Data for Figure 1 [file EMMM-10-e8926-s003.pdf]
