# Supplementary material for: Early auto‐immune targeting of photoreceptor ribbon synapses in mouse models of multiple sclerosis
Source: EMBO Mol Med. 2018 Sep 28;10(11):e8926. doi: 10.15252/emmm.201808926 (PMC6220320; doi:10.15252/emmm.201808926)

Figure 4B

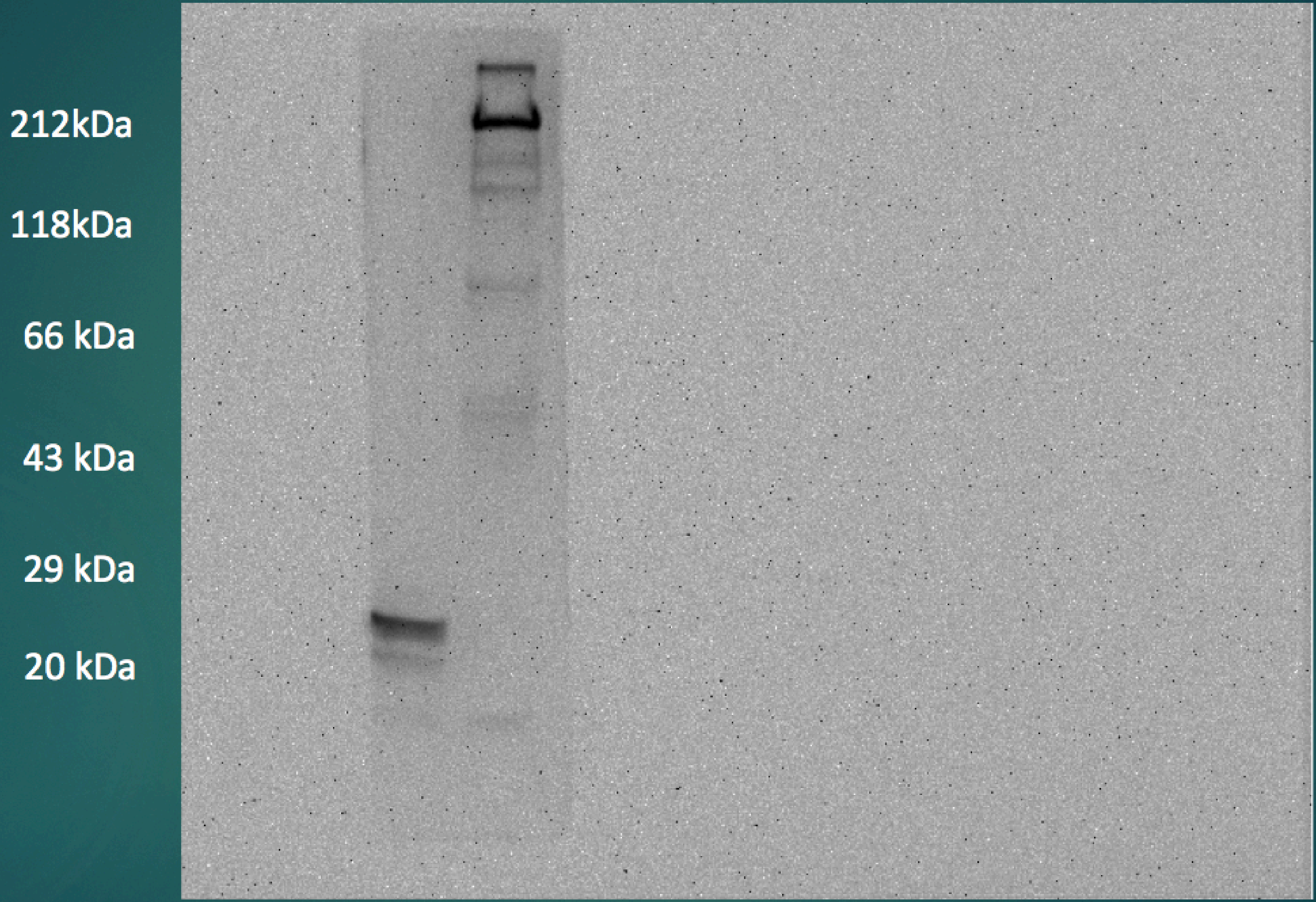

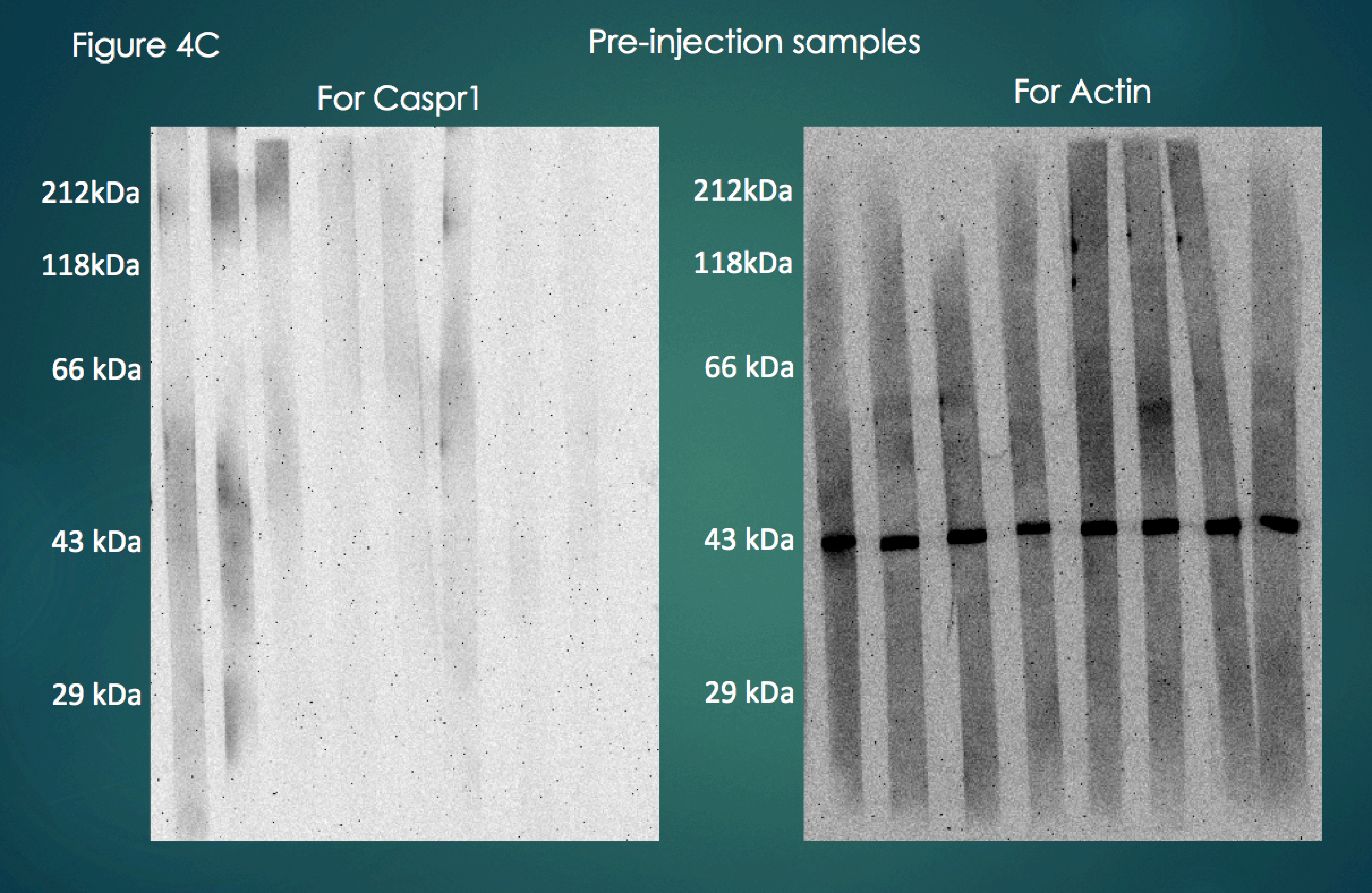
Figure 4C
Pre-injection samples
For Caspr1
For Actin
212kDa
118kDa
66 kDa
43 kDa
29 kDa
212kDa
118kDa
66 kDa
43 kDa
29 kDa

Figure 4D

Post injection samples

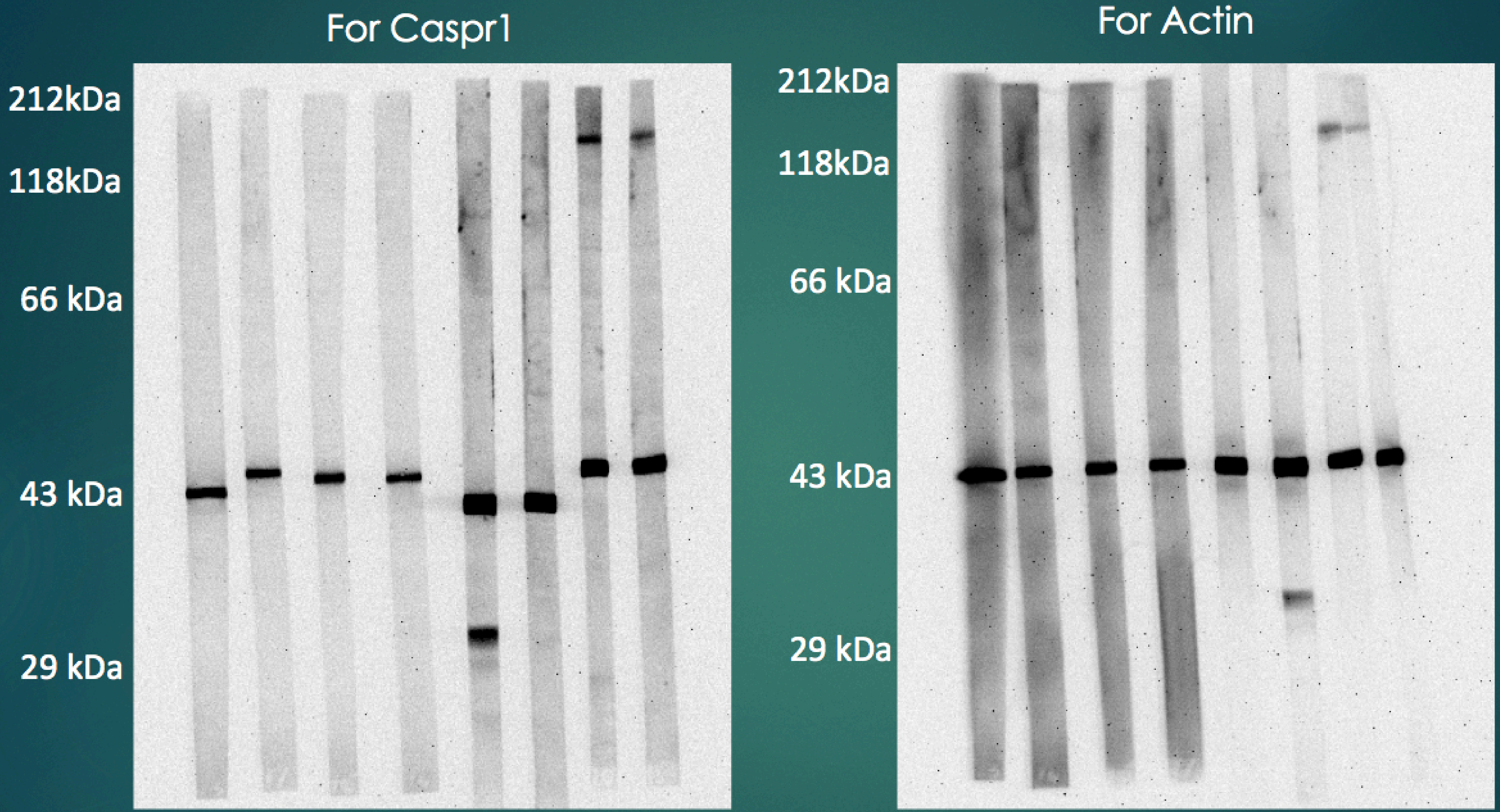

Supplement: Supplementary file 5 — Source Data for Figure 4 [file EMMM-10-e8926-s004.pdf]
